# Overweight/Obesity and Respiratory and Allergic Disease in Children: International Study of Asthma and Allergies in Childhood (ISAAC) Phase Two

**DOI:** 10.1371/journal.pone.0113996

**Published:** 2014-12-04

**Authors:** Gudrun Weinmayr, Francesco Forastiere, Gisela Büchele, Andrea Jaensch, David P. Strachan, Gabriele Nagel, the ISAAC Phase Two Study Group

**Affiliations:** 1 Institute of Epidemiology and Medical Biometry, Ulm University, Ulm, Germany; 2 Department of Epidemiology, Lazio Regional Health Service, Rome, Italy; 3 Division of Community Health Sciences, St. Georges’, University of London, London, United Kingdom; University of Modena and Reggio Emilia, Italy

## Abstract

**Background:**

Childhood obesity and asthma are increasing worldwide. A possible link between the two conditions has been postulated**.**

**Methods:**

Cross-sectional studies of stratified random samples of 8–12-year-old children (n = 10 652) (16 centres in affluent and 8 centres in non-affluent countries) used the standardized methodology of ISAAC Phase Two. Respiratory and allergic symptoms were ascertained by parental questionnaires. Tests for allergic disease were performed. Height and weight were measured, and overweight and obesity were defined according to international definitions. Prevalence rates and prevalence odds ratios were calculated.

**Results:**

Overweight (odds ratio = 1.14, 95%-confidence interval: 0.98; 1.33) and obesity (odds ratio = 1.67, 95%-confidence interval: 1.25; 2.21) were related to wheeze. The relationship was stronger in affluent than in non-affluent centres. Similar results were found for cough and phlegm, rhinitis and eczema but the associations were mostly driven by children with wheeze. There was a clear association of overweight and obesity with airways obstruction (change in FEV1/FVC, −0.90, 95%-confidence interval: −1.33%; −0.47%, for overweight and −2.46%, 95%-confidence interval: −3.84%; −1.07%, for obesity) whereas the results for the other objective markers, including atopy, were null.

**Conclusions:**

Our data from a large international child population confirm that there is a strong relation of body mass index with wheeze especially in affluent countries. Moreover, body mass index is associated with an objective marker of airways obstruction (FEV1/FVC) but no other objective markers of respiratory and allergic disorders.

## Introduction

The prevalence of obesity in childhood is increasing in many countries worldwide. Secular trends of increased obesity and asthma prevalence in adults and children during the past decades have led to a debate about potential links between both conditions. Suggested mechanisms to explain this association includes mechanical, lifestyle, dietary, immunological, hormonal, and common genetic factors [1].

There is evidence from cross-sectional studies [2], [3] that obesity is associated with asthma in childhood [4]–[6]. Prospective cohort studies show associations between obesity and incidence [7], [8] and persistence of asthma [9]. In a cohort study, overweight children had higher risk of asthma symptoms and bronchial hyper-responsiveness (BHR) at 8 years [10]. Although there is evidence of a link between obesity and asthma in children from predominantly western populations, little is known about less affluent regions and the under-lying mechanisms [11]. A recent report from the International Study of Asthma and Allergies in Childhood (ISAAC) Phase Three, a cross-sectional study, revealed associations between overweight and obesity and symptoms of asthma and eczema but not rhinoconjunctivitis [12]. However, no systematic evaluation of the association of objectively measured weight and height, with questionnaire reports together with objective measures of allergy and lung function has been available.

Despite the growing research over the obesity/asthma relationship, little is known about worldwide variation in the relationship of excess body mass index (BMI) and the prevalence of respiratory symptoms and allergic disease, and even less regarding objective markers for allergy and respiratory function. The ISAAC Phase Two study is ideally suited to address these issues because it includes a large number of children in geographically and economically diverse regions with data on various objective markers, and measured BMI in addition to a range of reported symptoms.

## Methods

### Study populations and field work

The methods of ISAAC Phase Two have been described in detail elsewhere [13]. Briefly, random samples of at least 10 schools from defined geographical areas were chosen and children (n>1 000 per centre) attending classes with a majority of 9–11-year-olds were invited to participate. Standardized parental questionnaires were used. In three countries (Ghana, Brazil and India) the questions were posed by trained interviewers because illiteracy was common.

The ISAAC Phase Two methodology allowed objective measurements to be performed either in the full sample (option A) or in random subsamples of children, generally stratified by wheeze (option B) [13]. Most centres invited all children to participate in the skin prick testing, while blood sampling, BHR tests and anthropometric measurements were carried out mostly in stratified random subsamples of children with and without reports of wheeze in the past year (targeting 100 per centre in each stratum).

Fuller details of the skin examination, lung function measurements and BHR, total immunglobulin E (IgE) measurements and skin prick tests to six aeroallergens (*Dermatophagoides pteronyssinus*, *D. farinae*, cat dander, *Alternaria tenuis*, mixed tree pollen and mixed grass pollen) have been published elsewhere [13] and can be found at http://isaac.auckland.ac.nz/phases/phasetwo/phasetwo.html.

### Symptoms data

Standardized parental questionnaires, including detailed questions on the occurrence and severity of symptoms of asthma (wheeze), rhinitis (with and without conjunctivitis) and flexural eczema were administered. In this context, wheezing is regarded as indicator symptom for asthma. These were identical to those used in ISAAC Phase One for parents of children aged 6–7 years [13]. In addition, in many (but not all) centres, questions about cough and phlegm were asked (http://isaac.auckland.ac.nz/phases/phasetwo/phasetwo.html and Online-Repository in File S1).

### Assessment of adiposity

Weight and height were measured without shoes and BMI was calculated. We used the age and sex specific BMI cut points for overweight and obesity derived from an international data set by Cole et al [14]. These cut points are based on age and sex specific percentile curves that were shown to correspond to the adult cut points of 25 and 30 kg/m^2^ for overweight and obesity, respectively. This procedure has the advantage of being independent of the prevalence of obesity in the individual study centres, thereby enabling their comparison and combining of results.

### Statistical analysis

Prevalence, population means and linear and logistic regression for health outcomes were calculated with the SURVEY-procedures of SAS (V9.2) using the appropriate weighting and variance estimation to account for stratified subsampling [15] where necessary. Total IgE as an outcome was dichotomized at the median (4.17 kU/l). Separate regression models were fitted for each centre and combined estimates of the effect estimates were derived using random effects meta-analysis [16]. Associations are presented as odds ratios (OR) or change in the parameter of interest. Forced expiratory volume in one second (FEV1) and forced vital capacity (FVC) as measured during spirometry were used in the statistical analysis, adjusting for age, gender and height, rather than using predicted values which may not be applicable in a large global setting with different child populations [17].

Potential confounders were tested by including them one by one in the centre-specific models and only those that resulted in a notable (10% or greater) change of the combined estimate were retained. The potential confounders included sex, age, diet (fruit and vegetable intake), Mediterranean diet, physical activity, reported parental allergic disease, maternal education, birth weight, breastfeeding, maternal smoking in pregnancy, anybody smoking in the child’s home, and damp spots or moulds in the child’s home. Based on the change-in-parameter criterion, only sex was retained in the fully adjusted model. Adjustment with physical activity and diet did not result in any change of the estimates but reduced the study population by about half. Results using adjustment for all other tested factors are presented in table S2 in File S2. Models for lung function (FEV1, FVC) were adjusted for age, sex and height.

The influence of potential effect modifiers was investigated by performing stratified centre-specific analyses, calculating the combined effect for each stratum and evaluating the difference between strata-specific estimates. Due to small cell counts in some centres in specific strata, the number of centres contributing to the stratum-specific estimates may differ from the number of centres in the corresponding unstratified analyses.

Centres classified by the World Bank as ‘high income countries’ (i.e. gross national income (GNI) per capita per year in 2001 ≧ 9 200 US $) were combined in a group called ‘affluent countries’ and the remaining centres in a group called ‘non-affluent countries’ [18].

### Ethics statement

All centres obtained approval by local ethics committees and investigators were trained in one location to assure comparable data quality. Almeria, Cartagena, Madrid, Valencia (all Spain): Ethics Committee of the “12 de Octubre” Hospital in Madrid; Tirana (Albania): National Ethics Committee; Tallinn (Estonia): The Medical Research Ethics Committee of Estonian Institute of Experimental and Clinical Medicine; Creteil (France): Comite Consultatif de Protection des Personnes dans la recherché biomedicale- Marseille 2; Dresden, Munich (both Germany): Ethics Committee of the University of Münster; Athens, Thessaloniki (both Greece): Hippokration General Hospital Ethical Committee; Rome (Italy): Ethic Committee of catholic University, Rome; Reykjavik (Iceland): National Bioethics Committee; Utrecht (Netherlands): Medical Ethical Committee of Wageningen University; Tromso (Norway): Regional Medical Ethics Committeee Norwegian Data Inspectorate; Linkoeping, Oestersund (both Sweden): Ethics committee at Linköping and Umea University; Ankara (Turkey): Ethics committee of Hacettepe University Faculty of Medicine; Ethics committee of the Turkish Ministry of Health; West Sussex (UK): Local Research Ethics Committee for Mid-Downs Health Authority; Hawkes Bay (New Zeland): Hawkes Bay Ethics Committee; Hong Kong (China): Ethics Committee of the Chinese University of Hong Kong; Beijing (China): Ethics Committee of Capital Institute of Paediatrics, Beijing; Guangzhou (China): Ethics Committee of Guangzhou Institute of Respirating Diseases; Kintampo (Ghana): London school of Hygiene and Tropical Medicine Ethics Committee; Mumbai (India): Jaslok Hospital; Uruguaiana (Brazil): Comite de Etica e Pesquisa da Pontificia Universidade Catolica do RGS; Pichincha (Ecuador): Saludesa/Hospital Pedro Vicente Maldonado Ad Hoc Ethics Committee; Tbilisi (Georgia): Bioethics National Counsil of Ministry of Labor, Health and Social Affairs of Georgia; Ramallah (Palestine): Palestinian Ministry of education; Palestinian Ministry of health; United Nations Relief and works Agency (UNRWA) school education dept.; Riga (Latvia): Ethics Committee of Riga Stradins University. The international coordination and collaboration has been approved by the ethics committees of the universities of Münster and Ulm, Germany [13]. Participation was voluntary and written consent from the parents was obtained for each child.

## Results

Table 1 presents mean BMI and the prevalence of overweight/obesity and the 12-months prevalence of wheeze for each study centre. An expanded version of the table, including all health-related outcomes, is included in Table S1 in File S2. Mean BMI was highest in Almeria (Spain) with 21 kg/m^2^ and lowest in Mumbai (India) with 15 kg/m^2^. The percentage of overweight children (excluding obese children) ranged from 0.6% in Kintampo (Ghana) to 38.7% in Uruguaiana (Brazil), and the percentage of obese children from 0 in Kintampo and Mumbai to 22.1% in Almerìa. Within Europe, there was a gradient with children from the Mediterranean region being more frequently obese. Wheeze was most frequent in Brazil (25.6%) and least prevalent in Athens, Greece (5.6%).

**Table 1:** Description of the study population: estimates referring to the full sample mean and prevalence are reported (implying appropriate weighting for stratified subsamples).

Centre	N	Age	BMI		Overweight		Obese		Wheeze
		Mean (95%-CI)	Mean (95%-CI)	N	% (95%-CI)	N	% (95%-CI)	N	% (95%-CI)
Brazil, Uruguaiana$	953	9.63 (9.58;9.68)	19.8 (19.6;20.0)	368	38.7 (35.6;41.8)	105	11.1 (9.1;13.1)	245	25.6 (23.7;27.6)
Estonia, Tallinn*	241	10.09 (10.05;10.13)	17.8 (17.5;18.1)	37	15.8 (10.9;20.8)	7	2.8 (0.6;5.0)	55	8.4 (6.7;10.2)
Georgia, Tbilisi*	169	10.41 (10.31;10.50)	18.8 (18.2;19.3)	31	20.1 (13.3;27.0)	12	6.4 (2.3;10.5)	54	9.2 (7.4;11.1)
Germany, Dresden§	694	9.87 (9.84;9.91)	17.4 (17.2;17.6)	100	14.4 (11.8;17.0)	21	3.0 (1.7;4.3)	52	7.9 (6.9;8.8)
Germany, Munich§	886	9.55 (9.51;9.59)	17.9 (17.7;18.1)	168	19.0 (16.4;21.5)	40	4.5 (3.1;5.9)	75	8.3 (7.3;9.2)
Ghana, Kintampo*	241	10.37 (10.28;10.46)	16.1 (16.0;16.3)	1	0.6 (0;1.7)	0	0	81	6.4 (5.1;7.7)
Greece, Athens*	193	9.79 (9.72;9.85)	19.9 (19.4;20.5)	54	27.7 (21.0;34.4)	30	15.4 (10.0;20.9)	37	5.6 (4.2;7.1)
Greece, Thessaloniki*	211	9.74 (9.66;9.82)	20.2 (19.7;20.6)	73	35.8 (28.4;43.3)	40	15.0 (9.6;20.4)	73	8.4 (6.7;10.1)
India, Mumbai*	119	9.77 (9.63;9.92)	15.0 (14.6;15.5)	7	4.9 (0.6;9.2)	0	0	33	6.1 (4.9;7.3)
Italy, Rome$	1307	10.02 (9.99;10.04)	19.4 (19.2;19.6)	402	30.8 (28.3;33.3)	137	10.5 (8.8;12.1)	103	7.9 (6.5;9.4)
Latvia, Riga§	156	10.54 (10.45;10.63)	18.0 (17.5;18.4)	20	12.8 (7.5;18.1)	3	1.9 (0;4.1)	16	6.9 (5.3;8.6)
Netherlands, Utrecht$	2638	9.50 (9.45;9.54)	17.8 (17.7;17.9)	413	15.7 (14.3;17.0)	98	3.7 (3.0;4.4)	238	8.7 (7.8;9.6)
New Zealand, Hawkes Bay*	222	10.76 (10.69;10.83)	19.8 (19.3;20.3)	52	22.4 (16.1;28.7)	25	9.5 (5.2;13.7)	111	21.9 (19.7;24.1)
Norway, Tromso*	637	9.94 (9.89;10.00)	17.9 (17.7;18.1)	106	16.0 (13.2;18.9)	25	3.4 (2.1;4.8)	131	14.0 (12.9;15.2)
Palestine, Ramallah*	216	9.73 (9.54;9.91)	17.8 (17.4;18.2)	25	12.2 (7.6;16.8)	10	4.0 (1.4;6.6)	43	8.8 (7.6;9.9)
Spain, Almeria*	208	10.26 (10.15;10.37)	21.0 (20.4;21.7)	65	33.1 (25.2;41.1)	45	22.1 (15.1;29.1)	105	15.5 (13.4;17.7)
Spain, Cartagena*	160	9.53 (9.44;9.63)	18.6 (18.0;19.2)	42	21.7 (14.2;29.2)	17	10.2 (4.6;15.8)	70	11.9 (10.2;13.6)
Spain, Madrid*	408	9.21 (9.15;9.27)	19.3 (18.9;19.6)	114	27.5 (23.1;31.9)	59	14.7 (11.1;18.2)	80	11.6 (9.6;13.7)
Spain, Valencia*	192	9.32 (9.22;9.42)	19.3 (18.8;19.7)	68	35.4 (28.5;42.3)	20	10.4 (6.0;14.8)	28	9.1 (7.6;10.7)
Sweden, Linkoeping*	181	10.72 (10.61;10.82)	19.4 (18.9;19.9)	42	23.6 (16.5;30.7)	14	5.6 (2.0;9.2)	59	7.9 (6.2;9.7)
Sweden, Oestersund*	278	10.70 (10.60;10.80)	18.7 (18.3;19.0)	49	15.5 (10.6;20.4)	17	3.8 (1.4;6.2)	109	10.2 (8.5;12.0)
Turkey, Ankara*	342	9.09 (9.03;9.14)	17.7 (17.4;18.1)	52	17.0 (12.0;22.0)	21	6.9 (3.5;10.3)	160	10.9 (9.8;12.0)

$ Full sample;

*Stratified random subsample: 100 children with and 100 children without wheeze were invited.

§ Random sample: a random sample without stratification by wheeze was invited.

**Figure 1:**
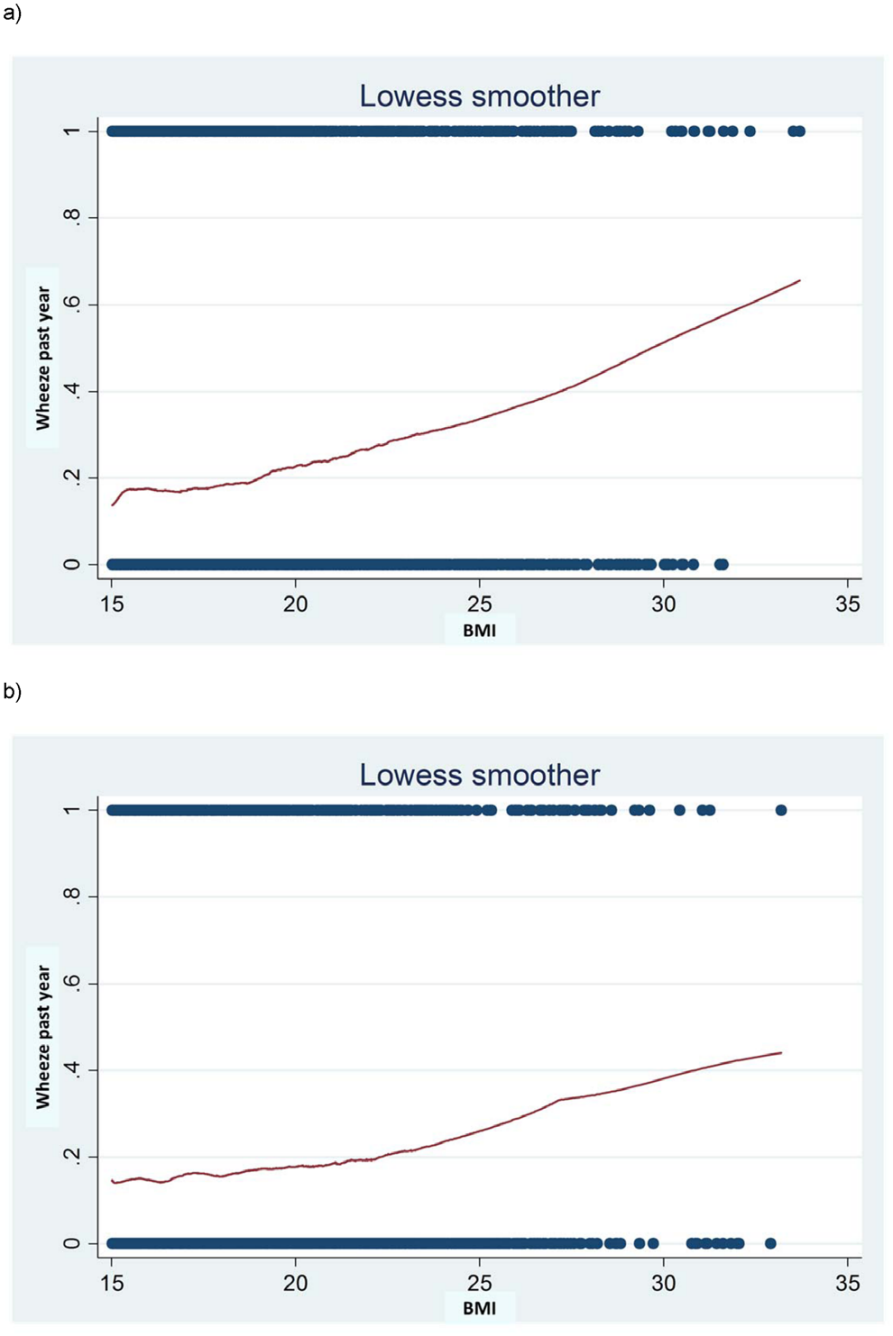
Lowess plots i.e. smoothed curves of the average proportion of wheeze in the past year in relation to BMI (a: boys; b: girls).

Figure 1 shows the relation between BMI and proportion of wheeze in the past year including children from all centres. A monotonic increase of wheeze with increase in BMI was seen with no deviation from linearity. There was a slight indication for a stronger slope for boys than for girls. When we calculated combined ORs for all centres from meta-analysis, there were no statistically significant differences between boys and girls. We therefore report results for boys and girls combined, but also provide additional sex-specific results in Table S7 in File S2.

**Figure 2:**
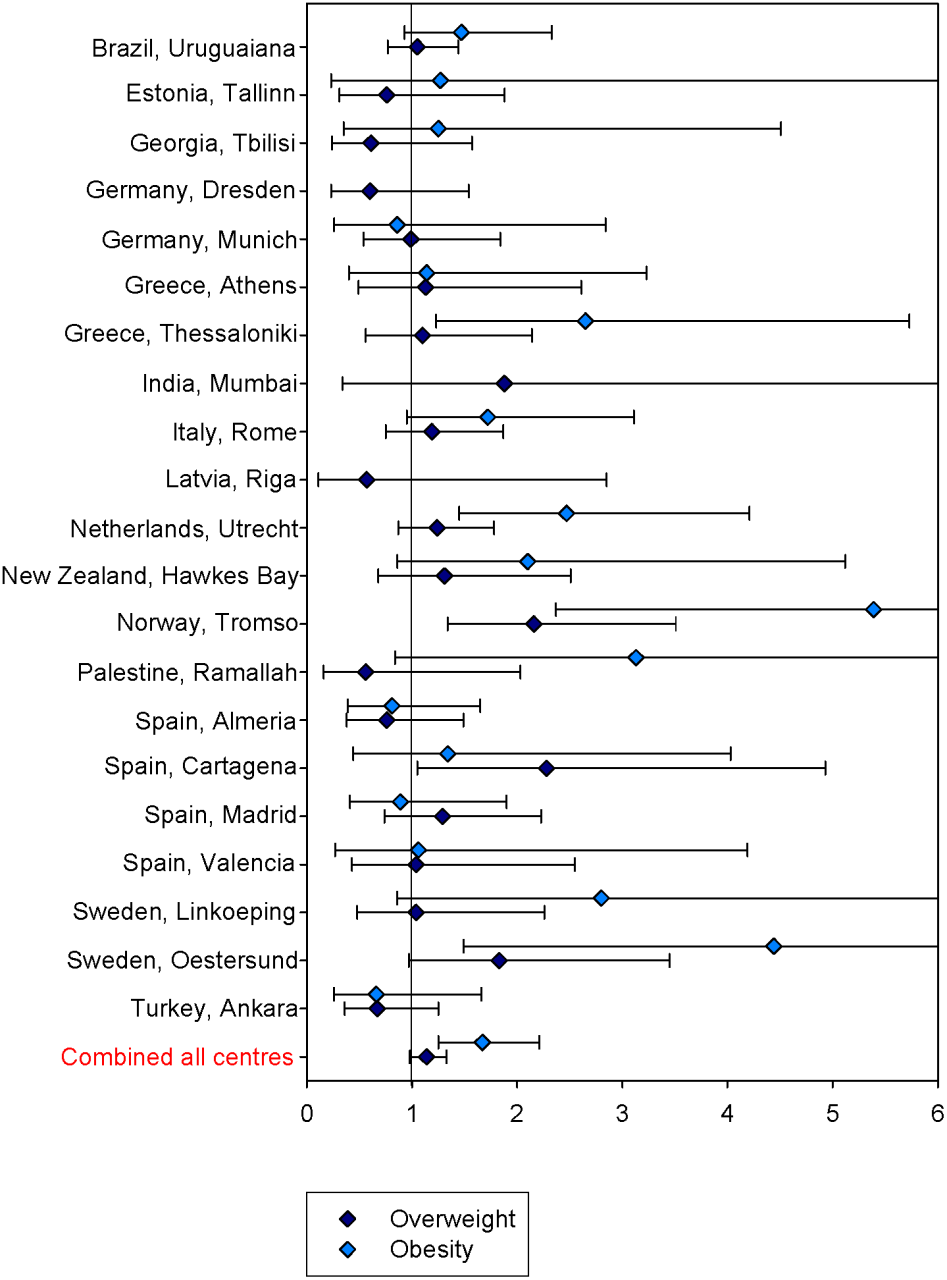
Odds ratios with 95%-confidence intervals (adjusted for sex) for the association of wheeze in the past year with overweight and obesity.

The combined OR for wheeze in the past year in relation to overweight and to obesity was 1.14 (95% confidence interval (CI): 0.98; 1.33) and 1.67 (95%-CI: 1.25; 2.21), respectively (Table 2). The greater part of the centres showed positive ORs with wheeze for overweight and even more so for obesity (Figure 2). There was a stronger association in affluent centres than in non-affluent centres (Tables 2 and 3). This difference between affluent and non-affluent centres was statistically significant for overweight (p = 0.024) where no association was seen in the non-affluent countries, but not for obesity (p = 0.267). Within affluent European centres, there was an indication of a stronger association with obesity among centres from North-Central Europe (2.84, 95%-CI: 1.67; 4.82) as opposed to Southern Europe, but much less so for overweight (1.31, 95%-CI: 0.91; 1.89, Table S3 in File S2).

**Table 2:** Association of wheeze, respiratory and allergy related outcomes and eczema symptoms with overweight and obesity: combined estimates from meta-analysis, adjusted for sex.

		Overweight			Obese		
		OR (95%-CI)	N	n*	OR (95%-CI)	N	n*
**Wheeze past year**	All centres	1.14 (0.98;1.33)	9658	21	1.67 (1.25;2.21)	7274	18
	Affluent	1.27 (1.08;1.50)	7623	14	1.80 (1.28;2.53)	5869	13
	Nonaffluent	0.86 (0.65;1.16)	2035	7	1.34 (0.91;1.98)	1405	5
**Wheeze with exercise**	All centres	1.24 (1.01;1.52)	7221	17	1.65 (1.19;2.29)	5995	16
	Affluent	1.33 (1.06;1.66)	5580	12	1.58 (1.04;2.38)	4801	12
	Nonaffluent	0.88 (0.47;1.63)	1641	5	1.64 (1.00;2.70)	1194	4
**Wheeze without exercise**	All centres	1.10 (0.80;1.52)	5359	13	1.20 (0.81;1.77)	4323	12
	Affluent	1.15 (0.76;1.73)	3870	9	1.12 (0.74;1.67)	3329	9
	Nonaffluent	0.98 (0.49;1.98)	1489	4	1.48 (0.31;6.99)	994	3
**Sleep disturbing wheeze**	All centres	1.36 (0.99;1.86)	7447	15	2.15 (1.21;3.82)	6338	13
	Affluent	1.30 (0.77;2.18)	5981	11	2.27 (1.16;4.44)	5566	11
	Nonaffluent	1.44 (0.93;2.21)	1466	4	2.04 (0.46;9.09)	772	2
**Dry cough at night**	All centres	1.13 (1.00;1.29)	9498	21	1.28 (1.07;1.54)	7729	19
	Affluent	1.21 (1.05;1.40)	7487	14	1.34 (1.08;1.65)	6341	14
	Nonaffluent	0.93 (0.73;1.19)	2011	7	1.32 (0.63;2.79)	1388	5
**Woken with shortness of breath**	All centres	1.01 (0.84;1.20)	4221	14	1.35 (0.89;2.04)	2949	11
	Affluent	1.06 (0.76;1.48)	2417	8	1.18 (0.62;2.24)	1816	7
	Nonaffluent	0.94 (0.59;1.50)	1804	6	1.70 (0.80;3.61)	1133	4
**Severe wheeze**	All centres	1.29 (1.02;1.64)	8525	20	1.69 (1.19;2.38)	6599	19
	Affluent	1.37 (1.03;1.80)	6968	14	1.99 (1.31;3.03)	5338	13
	Nonaffluent	0.93 (0.43;2.04)	1557	6	1.17 (0.73;1.88)	1261	6
**Severe wheeze among wheezers**	All centres	1.14 (0.87;1.50)	1562	18	1.08 (0.77;1.51)	1298	17
	Affluent	1.09 (0.81;1.47)	1066	13	1.08 (0.71;1.65)	878	12
	Nonaffluent	1.12 (0.57;2.20)	496	5	1.10 (0.59;2.07)	420	5
**BHR yes/no**	All centres	1.05 (0.88;1.26)	5056	19	1.09 (0.81;1.47)	3916	16
	Affluent	1.13 (0.93;1.36)	4135	14	1.10 (0.80;1.50)	3515	14
	Nonaffluent	0.57 (0.32;1.01)	921	5	1.05 (0.42;2.63)	401	2
**Skin prick test**	All centres	1.04 (0.91;1.18)	7891	21	1.13 (0.91;1.42)	6194	18
	Affluent	1.03 (0.90;1.19)	5954	14	1.07 (0.87;1.33)	5004	14
	Nonaffluent	1.05 (0.77;1.44)	1937	7	1.49 (0.61;3.67)	1190	4
**Total IgE** $	All centres	0.95 (0.80;1.12)	4451	15	1.07 (0.82;1.39)	3709	15
	Affluent	0.97 (0.81;1.15)	4144	13	1.07 (0.81;1.39)	3571	14
	Nonaffluent	0.76 (0.40;1.44)	307	2	Only one centre left		
**Rhinitis**	All centres	0.99 (0.88;1.11)	9522	21	1.19 (0.90;1.56)	7746	19
	Affluent	0.95 (0.83;1.09)	7506	14	1.07 (0.79;1.45)	6358	14
	Nonaffluent	1.11 (0.87;1.41)	2016	7	1.71 (0.94;3.11)	1388	5
**Rhinitis with wheeze**	All centres	1.10 (0.93;1.32)	7161	21	1.73 (1.26;2.38)	5379	18
	Affluent	1.12 (0.91;1.38)	5752	14	1.65 (1.10;2.46)	4426	13
	Nonaffluent	1.06 (0.76;1.48)	1409	7	1.99 (1.24;3.19)	953	5
**Rhinitis without wheeze**	All centres	0.99 (0.86;1.13)	7878	21	1.13 (0.85;1.51)	6243	18
	Affluent	0.96 (0.83;1.12)	6408	14	1.03 (0.75;1.42)	5274	13
	Nonaffluent	1.07 (0.80;1.43)	1470	7	1.56 (0.79;3.08)	969	5
**Reported eczema past year**	All centres	1.19 (1.02;1.39)	9376	20	1.45 (0.94;2.24)	7471	18
	Affluent	1.26 (1.06;1.50)	7494	14	1.62 (0.95;2.79)	6236	13
	Nonaffluent	0.98 (0.71;1.35)	1882	6	0.84 (0.49;1.47)	1235	5
**Reported eczema without wheeze**	All centres	1.18 (0.99;1.41)	7667	19	1.24 (0.88;1.75)	5900	15
	Affluent	1.26 (1.03;1.54)	6290	13	1.34 (0.93;1.92)	5260	12
	Nonaffluent	0.94 (0.64;1.37)	1377	6	1.01 (0.35;2.94)	640	3
**Eczema by examination**	All centres	1.36 (0.98;1.87)	6348	14	1.18 (0.70;2.00)	4292	11
	Affluent	1.39 (0.98;1.98)	5505	10	1.19 (0.69;2.06)	4154	10
	Nonaffluent	1.06 (0.40;2.81)	843	4	Only one centre left		
**Examined eczema without wheeze**	all centres	1.27 (0.88;1.82)	5366	14	2.07 (1.03;4.17)	2656	7
	affluent	1.29 (0.85;1.96)	4802	10	2.25 (1.08;4.68)	2565	6
	nonaffluent	1.15 (0.39;3.40)	564	4	Only one centre left		
		Mean change (%) (95%-CI)	N	n*	Mean change (%) (95%-CI)	N	n*
**FEV1/FVC^&#^**	All centres	−0.90 (−1.33;−0.47)	5439	9	−2.46 (−3.84;−1.07)	4746	9
	Affluent	−0.94 (−1.43;−0.44)	4970	7	−1.59 (−2.31;−0.86)	4324	7
	Nonaffluent	−0.79 (−2.28;0.70)	469	2	−5.39 (−9.46;−1.31)	422	2
		Mean change (ml)	N	n*	Mean change (ml)	N	n*
**FEV1** &	All centres	80.84 (48.09;113.58)	7926	21	80.26 (52.79;107.74)	6544	19
	Affluent	61.34 (42.69;79.99)	6498	14	82.96 (49.28;116.64)	5586	14
	Nonaffluent	123.40 (37.86;208.95)	1428	7	53.80 (−38.70;146.30)	958	5
		Mean change (ml)	N	n*	Mean change (ml)	N	n*
**FVC** &	All centres	105.57 (84.57;126.56)	5494	9	172.05 (125.68;218.42)	4790	9
	Affluent	105.73 (83.97;127.49)	5023	7	157.17 (113.64;200.69)	4367	7
	Nonaffluent	103.30 (23.49;183.12)	471	2	265.25 (137.21;393.29)	423	2

*Number of centres.

$ Dichotomized at 4.17 kU/l.

& Adjusted for age, sex and height.

# Exclusion of children with difference of FEV1 and FVC>200 ml or difference = −12 000 ml.

**Table 3:** Association of overweight and obesity with wheeze and effect modification: OR with 95%-confidence intervals adjusted for sex.

	OR	N	n(centres)	OR	N	n(centres)	P-value for difference
	**Affluent countries**			**Nonaffluent countries**			
Overweight	1.27 (1.08;1.50)	7623	14	0.86 (0.65;1.16)	2035	7	0.024
Obese	1.80 (1.28;2.53)	5869	13	1.34 (0.91;1.98)	1405	5	0.27
	**Boys**			**Girls**			
Overweight	1.26 (1.05;1.53)	4801	20	0.98 (0.71;1.34)	4628	19	0.17
Obese	1.94 (1.45;2.60)	3768	18	1.47 (1.08;2.00)	3068	16	0.20
	**Atopics**			**Nonatopics**			
Overweight	1.22 (0.95;1.56)	2095	20	1.05 (0.87;1.27)	5328	20	0.35
Obese	1.65 (1.14;2.39)	1403	14	1.70 (1.08;2.66)	3537	16	0.92
	**Parental allergic disease**			**No parental allergic disease**			
Overweight	1.10 (0.86;1.41)	4753	18	1.21 (0.98;1.49)	4496	19	0.57
Obese	1.97 (1.49;2.62)	3757	17	1.48 (1.05;2.08)	3190	15	0.20
	**Parental asthma**			**No parental asthma**			
Overweight	1.18 (0.84;1.67)	1126	16	1.14 (0.96;1.36)	8078	20	0.86
Obese	1.88 (1.13;3.12)	772	12	1.65 (1.24;2.19)	6066	17	0.66
	**BHR**			**No BHR**			
Overweight	1.02 (0.73;1.44)	1126	18	1.12 (0.84;1.49)	3918	19	0.69
Obese	1.37 (0.82;2.28)	825	14	1.94 (1.19;3.16)	2607	14	0.34
	**Mother smoked in pregnancy**			**No maternal smoking in pregnancy**			
Overweight	1.21 (0.87;1.68)	1427	10	1.16 (0.98;1.37)	6873	19	0.85
Obese	1.93 (1.24;2.99)	1171	10	1.52 (1.19;1.94)	5063	16	0.35
	Fresh fruits intake*			**No fresh fruits intake**			
Overweight	1.20 (0.94;1.53)	4233	15	0.88 (0.52;1.49)	414	10	0.29
Obese	1.53 (1.04;2.25)	3227	13	1.35 (0.59;3.07)	252	7	0.78
	**Med score first tertile^§^**			**Med score second and third tertile**			
Overweight	1.33 (1.03;1.72)	1918	13	1.15 (0.81;1.64)	1841	13	0.51
Obese	1.50 (1.05;2.14)	1449	11	1.41 (0.95;2.10)	1380	11	0.82
	**Physical activity** +			**Physical activity^−^**			
Overweight	1.32 (1.01;1.74)	2656	13	0.93 (0.63;1.38)	817	11	0.15
Obese	1.69 (1.04;2.76)	2075	12	1.07 (0.61;1.88)	676	11	0.23
	**Urban^$^**			**Rural^$^**			
Overweight	1.08 (0.81;1.45)	3401	18	1.22 (0.91;1.62)	2095	15	0.58
Obese	1.24 (0.92;1.66)	2262	13	2.05 (1.22;3.45)	1470	13	0.10
	**Low birth weight^&^**			**Normal birth weight^&^**			
Overweight	1.65 (0.89;3.06)	417	11	1.09 (0.83;1.42)	2746	17	0.23
Obese	2.54 (0.86;7.50)	159	4	1.35 (0.82;2.23)	1996	14	0.30
	**High birthweight^&^**			**Normal birth weight^&^**			
Overweight	1.40 (1.08;1.82)	1816	15	1.09 (0.83;1.42)	2746	17	0.18
Obese	2.10 (1.49;2.98)	1508	15	1.35 (0.82;2.23)	1996	14	0.15

+ Physical activity: 2 times a week or more often; ^*^Fresh fruits are consumed 1 time per week or more often; ^§^Med.Score: higher scores for more frequent consumption of vegetables (raw green and cooked), fruit, fruit juice and fish, and lower scores for more frequent consumption of meat, burger and fizzy drinks (for details see File S1 and S2); ^&^Low birthweight: less than 2500 g, normal: 2500–3499 g, high: more than 3500 g; ^$^urban: suburban, with few parks or gardens or urban with no parks or gardens, rural: rural, open spaces or fields nearby or suburban, with many parks or gardens.

When looking at wheeze characteristics, we found that wheeze with exercise was statistically significantly associated with overweight and obesity, especially in affluent centres, whereas wheeze in the absence of exercise was not. Children reporting more often dry cough at night and those woken with tightness of chest were more often obese. Effects were most pronounced in North-Central Europe (Table S3 in File S2). There was no strong evidence for an association with children woken with shortness of breath. Also, among wheezers, there was no indication of more severe wheeze in overweight or obese children.

Except for affluence in overweight children, none of the tested potential effect modifiers gave a statistically significant result (Table 3). For some factors, there was a slight indication with p-values ≤0.2 and with a statistically significant positive associations only in one stratum, namely in physically active children, children with high birth weight and boys. For rural and urban surroundings, effect estimates were both positive but higher for rural (p = 0.1).

None of the three objective measures of BHR, skin prick tests, and total IgE was conclusively associated with overweight or obesity (Table 2). ORs were close to one for skin prick test and total IgE, and, while there was an indication of a positive association with BHR in Northern-Central Europe, an inverse association with overweight was observed in non-affluent centres. There was a statistically significantly lower FEV1/FVC in relation with overweight and obesity in affluent centres and even more so with obesity in non-affluent centres. FEV1 and FVC adjusted for age, sex and height, on the contrary, showed an increase of approximately 80 ml (overweight and obese) for FEV1 and 106 ml (overweight) and 172 ml (obese) for FVC.

Regarding other allergic disease symptoms (Table 2 and Table S4 in File S2), rhinitis was not associated with overweight and obesity. For eczema (reported and examined), there was an association with overweight and obesity in affluent countries but not in non-affluent countries. The figures for examined eczema in obese children should be viewed with caution due to the reduced number of centres (and children) in the analysis. Looking at respiratory symptoms (Table 4 and Table S5 in File S2), the association of coughed up phlegm with overweight and obesity was observed mostly when occurring in the absence of a cold or frequently among children from affluent countries. All associations were moderately stronger in children who also reported wheeze at the same time (Table S6 in File S2).

**Table 4:** Association of cough and phlegm with overweight and obesity: combined estimates from meta-analysis adjusted for sex.

	Overweight			Obese		
	OR (95%-CI)	N	n*	OR (95%-CI)	N	n*
**Coughed up phlegm with colds**						
All centres	1.11 (0.94;1.30)	7646	17	1.26 (0.96;1.66)	6251	16
Affluent	1.19 (1.00;1.43)	5824	11	1.32 (0.95;1.83)	4908	11
Nonaffluent	0.96 (0.77;1.21)	1822	6	0.86 (0.37;1.99)	1343	5
**Coughed up phlegm with colds without wheeze**						
All centres	1.03 (0.84;1.26)	6255	17	1.25 (0.98;1.59)	4949	15
Affluent	1.06 (0.82;1.38)	4946	11	1.27 (0.95;1.70)	4158	11
Nonaffluent	0.94 (0.72;1.22)	1309	6	1.11 (0.71;1.74)	791	4
**Coughed up phlegm without colds**						
All centres	1.17 (0.98;1.40)	7541	17	1.64 (1.15;2.34)	5840	14
Affluent	1.30 (0.99;1.71)	5755	11	1.73 (1.10;2.71)	4859	11
Nonaffluent	1.12 (0.80;1.58)	1786	6	1.13 (0.38;3.30)	981	3
**Coughed up phlegm without colds without wheeze**						
All centres	1.07 (0.83;1.37)	5375	13	1.86 (1.32;2.63)	4418	10
Affluent	1.10 (0.78;1.56)	4176	8	1.88 (1.27;2.80)	3846	8
Nonaffluent	1.13 (0.63;2.05)	1199	5	1.78 (0.76;4.17)	572	2
**Congested in chest/coughed up phlegm frequently** §						
All centres	1.44 (1.15;1.79)	6935	15	2.28 (1.70;3.07)	5482	13
Affluent	1.70 (1.29;2.25)	5363	10	2.53 (1.62;3.93)	4546	10
Nonaffluent	0.91 (0.49;1.68)	1572	5	2.01 (1.26;3.22)	936	3
**Congested in chest/coughed up phlegm frequently** § **without wheeze**						
All centres	1.52 (1.00;2.30)	4947	11	2.71 (1.76;4.17)	3824	9
Affluent	1.87 (1.24;2.82)	3915	7	2.93 (1.55;5.52)	3270	7
Nonaffluent	0.85 (0.46;1.60)	1032	4	2.65 (1.28;5.50)	554	2

*Number of centres.

§ On 4 or more days a week for as much as 3 months a year.

## Discussion

This large international multi-centre study, including both affluent and non-affluent countries, provides evidence that excess weight is associated with asthmatic symptoms as well as eczema, and rhinitis in combination with asthmatic symptoms. No association was observed for objective measures of allergic disease such as BHR, skin prick test, or total IgE. BMI was inversely associated with FEV1/FVC, an indicator of airway obstruction.

In our study, we found a linear association between BMI and wheeze indicating a dose-response relationship. However, other authors observed a U-shaped BMI-asthma relationship, when applying BMI categories [19]. We found a stronger association with wheeze for overweight in affluent countries and within Europe an indication for a stronger association with obesity in Northern-Central than in Southern European centres. A dose response pattern was also found for coughed up phlegm. Stronger associations due to more power could be attributed to higher prevalence of weight excess in western countries [20].

A lower FEV1/FVC was observed in overweight and obese children from both, affluent and non-affluent countries. However, it should be acknowledged that data on FVC was only available in a subset. Literature examining the association between obesity and lung function is conflicting. Height has been identified as the important independent predictor of spirometric variables and the therefore resulting collinearity of FEV1 and FVC with BMI limits the interpretation of these parameters on their own [21]. Our observation that the effects of obesity on FEV1/FVC are related to an increase in FVC, rather than a decrease in FEV1 has therefore to be interpreted with caution. In a certain proportion of children, BMI may reflect increased muscular mass and be correlated with higher lung volumes and therefore influence the observed relationship with FVC and FEV1, respectively [22], while this effect is cancelled out in the ratio between them. Nevertheless, the reduction of FEV1/FVC but no increase of BHR could suggest that overweight and obesity are not related to the common asthma phenotype associated with BHR. Indeed, there is cumulating evidence that asthma is a heterogeneous disease with different phenotypes and potential different pathological mechanisms [23], [24]. Obesity and asthma are believed to share common genetic determinates [25] and it has been suggested that obesity results in a distinct asthma phenotype [26], characterized by more severe disease, with increased exacerbation, poorer asthma control and steroid responsiveness. In our study, wheeze with exercise, dry cough at night, woken at night with tightness of chest and coughed up phlegm without a cold were found more frequently among overweight and obese children. This could reflect differential patterns of asthma phenotypes between normal weight as opposed to overweight/obese children. The stronger association of wheeze with overweight/obesity in affluent countries, in particular countries from Northern-Central Europe, may possibly reflect an increased prevalence of an obesity-related asthma phenotype.

In our study, the association between excess weight and wheeze was not related to atopic status. This contradicts some previous reports on stronger associations for non-atopic asthma, but is in line with the observations that have not found an effect modification by atopy [2]. Consistent with the literature, we found no evidence that overweight and obesity is associated with allergic sensitization [27]. Our observation of mixed results for BHR in affluent countries and non-affluent countries fits with the heterogeneous results from former publications [10], [19], [28]. For reported eczema we observed increased prevalence among obese children with asthma symptoms in agreement with cross-sectional and longitudinal studies [12], [29], and the data for clinical examination of eczema seem to corroborate these results.

We did not find strong evidence for an association between overweight and obesity and allergic disease, since only rhinitis in combination with wheeze and none of the objective measures for allergy was associated with overweight and obesity. This is in line with other studies including objective markers [29] suggesting a non-eosinophil inflammatory mechanism is associated with asthma. Obesity is thought to be linked with asthma by obesity-related systemic inflammation, which could promote exaggerated responses to environmental triggers [1]. Obesity is associated with low-grade inflammation and adipokines, which have been found to be related to asthma symptoms [30].

In our study, the association of overweight and obesity with asthma symptoms did not differ between girls and boys. In previous cross-sectional and longitudinal studies, gender-differences have been reported in some [8], [11], [29] but not in other studies [2], [9], [12], [27], [29].

We observed stronger associations between overweight and asthma in affluent and in particular Northern-Central European countries than in non-affluent countries. Differences of asthma prevalence according to affluence status are well established [31]. Lifestyle and standards of living are suggested to contribute to these disparities, and may also reflect partly different structures of residual confounding as indicated by former ISAAC II publications on breastfeeding [32], diet [33], infections [34], and dampness [35]. The existence of different asthma phenotypes [24] may also contribute to the observed pattern. Epidemiological studies in adults and also children revealed ethnic differences in fat deposits [36] which can also apply in different relationships of the fat mass and fat free mass in children. The differences could contribute to geographic changes in the associations of BMI with asthma and allergic diseases.

Several possible limitations of the study need to be considered. Parental reporting on disease symptoms, in particular for asthma, could have been influenced by obesity, since in adults over-diagnosis of asthma has been matter of concern [30]. In our study, however, the positive associations between obesity and wheeze were also present when considering lung function. Residual confounding could have affected the associations. However, by further adjustment for dietary factors such as fruit and vegetable intake, Mediterranean diet score or physical activity the associations between obesity and asthma symptoms did not change. Other comorbid conditions related to obesity could have influenced the occurrence of asthma [37]. As we investigated a number of outcomes, we cannot exclude the possibility of a sporadic chance finding among the findings which are, however, quite consistent in their overall pattern. In large worldwide studies, a difficulty is that information may not be comparable across geographical regions and therefore covariates may not reflect exactly the same underlying confounders. This may have limited our ability to find and adjust for relevant confounders, so that we cannot exclude fully the possibility of residual confounding. On the other hand the presence of quite consistent associations across these geographical regions is reassuring, decreasing the probability that the overall result is due to residual confounding, only.

Further strengths of the study are the use of the standardized ISAAC questionnaires and methodology in all centres and the inclusion of non-affluent countries. Another strength is that BMI has been measured according to a standard protocol extending the work from ISAAC Phase Three which was largely based on reported values [12]. Furthermore, we were able to investigate standardized objective measurements for lung function and allergic disease in a large international study. However, due to the cross-sectional study design reverse causation cannot be completely excluded.

## Conclusion

Our observations in a large international child population strengthen previous reports that overweight and obesity are associated with wheeze and asthma in childhood as well as objective evidence of airways obstruction. No other objective markers of respiratory and allergic disorders were involved. The significance of these observations needs to be confirmed in prospective studies and experimental trials.

## Supporting Information

File S1 Contains detailed information of additional outcomes.
(DOC)

File S2 Additional Tables: **Table S1:** Mean and prevalence of investigated outcomes with 95% confidence interval. **Table S2:** Test for confounding: results from fully adjusted model including all potential factors tested. **Table S3:** Association of wheeze and related symptoms with overweight and obesity: combined estimates from meta-analysis, adjusted for sex. **Table S4:** Association of respiratory and allergy related outcomes and eczema with overweight and obesity, adjusted for sex. **Table S5:** Association of cough and phlegm with overweight and obesity: combined estimates from meta-analysis adjusted for sex. **Table S6:** Association of overweight and obesity with respiratory and allergy related outcomes and eczema in the absence/presence of wheeze: combined estimates with 95%-confidence intervals from meta-analysis adjusted for sex. **Table S7:** Association of respiratory and allergy related outcomes and eczema with overweight and obesity: analysis by sex.
(DOC)
