# Correction: Overweight/Obesity and Respiratory and Allergic Disease in Children: International Study of Asthma and Allergies in Childhood (ISAAC) Phase Two

**DOI:** 10.1371/journal.pone.0126678

**Published:** 2015-04-22

**Authors:** Gudrun Weinmayr, Francesco Forastiere, Gisela Büchele, Andrea Jaensch, David P. Strachan, Gabriele Nagel, the ISAAC Phase Two Study Group

Marianne van Hage is not included in the Acknowledgments. She should be included in the Phase Two Principal Investigators section of the Acknowledgments. Her affiliation is IgE analyses—Stockholm, Sweden.
