# Supplementary material for: Overweight/Obesity and Respiratory and Allergic Disease in Children: International Study of Asthma and Allergies in Childhood (ISAAC) Phase Two
Source: PLoS One. 2014 Dec 4;9(12):e113996. doi: 10.1371/journal.pone.0113996 (PMC4256390; doi:10.1371/journal.pone.0113996)
Supplement: File S1 — Contains detailed information of additional outcomes. (DOC) [file pone.0113996.s001.doc]

**Online Repository:**

**Overweight/obesity and respiratory and allergic disease in children: ISAAC Phase Two**

Gudrun Weinmayr^1^, Francesco Forastiere^2^, Gisela Büchele^1^, Andrea Jaensch^1^, David P Strachan^3^, Gabriele Nagel^1^; and the ISAAC Phase Two Study Group*

^1^ Institute of Epidemiology and Medical Biometry, Ulm University, Ulm, Germany;

^2^Department of Epidemiology, Lazio Regional Health Service, Rome, Italy;

^3^Division of Community Health Sciences, St. Georges’, University of London, London, United Kingdom;

* listed at the end of paper.

**Description of additional outcomes:**

*Questions regarding asthma severity:*

Wheeze was classified as severe if any of the following was reported: Four or more wheezing attacks; Wheeze disturbing sleep; Wheeze severe enough to limit speech.

*Questions regarding cough and phlegm:*

Coughed up phlegm without a cold: “In the last 12 months, has your child usually seemed congested in the chest or coughed up phlegm when he/she did not have a cold?” Yes/No

Coughed up phlegm frequently: “Does your child seem congested in the chest or chough up phlegm on most days (4 or more days a week) for as much as 3 months of the year?” Yes/No

*Skin exam for flexural eczema*

Children were physically examined for flexural eczema in the following 5 body areas: 1) around the eyes, 2) the neck, 3) in front of the elbows, 4) behind the knees, and 5) in front of the ankles. Participants were categorized as having flexural eczema if they had a typical erythematous rash with surface change (e.g. fine scaling, vesicles, oozing, crusting or lichenification) in any of the above mentioned flexural areas (<http://www.nottingham.ac.uk/dermatology/eczema/index.html>). All fieldworkers were first trained and then formally tested in the recognition of flexural eczema, using a manual and photographic images specifically developed for this purpose [1]; <http://www.nottingham.ac.uk/dermatology/eczema/index.html>).

*Bronchial Hyperresponsiveness*

Spirometry was performed according to ATS criteria [2] and was described elsewhere in detail [1]. A total of 9312 children underwent a bronchial hyperreactivity (BHR) test using a de Vilbiss Ultraneb 2000 nebuliser. Children inhaled a nebulised hyperosmolar saline solution (4.5 %) for increasing time periods (0.5, 1, 2, 4, and 8 minutes) [3]. Forced expiratory volume (FEV1) was measured 1 min after the end of each inhalation period, and the next challenge was performed after 3 min wash-out time. If the FEV1 fell 10-15 % below the baseline value, the previous exposure time was repeated. If, after two repetitions, the fall in FEV1 was still between 10% and 15%, the exposure time was also doubled. Bronchial challenge was stopped if either the FEV1 had fallen by 15% or more, or the total inhalation period of 15.5 minutes had been reached. In children with a baseline FEV1 of <75% of predicted value, no bronchial challenge was performed and an inhaled bronchodilator was administered. Children were considered BHR-positive if FEV1 had fallen by 15% compared to baseline, or increased by 25% or more after administration of a bronchodilator.

*Skin prick tests (SPTs)*

The SPTs were carried out according to a detailed protocol [1]. Six extracts of common aeroallergens (Dermatophagoides pteronyssinus, D. farinae, cat dander, Alternaria tenuis, mixed tree pollen and mixed grass pollen) produced by ALK (Horsholm Denmark) were used. The centres were encouraged to add allergens of local relevance. Additional allergens were tested in seventeen centres and include: Olive pollen, Parietaria officinalis, cockroach, dog, horse, Cladosporium herbarum, local pollen mixes and feather (in Turkey, [4]) and mixed moulds (in China). A positive skin reaction was defined as a wheal size ≥ 3mm, after subtraction of the negative control.

*Mediterranean Score*

Points were assigned for ‘low’ consumption (less than once per week; score 0), ‘regular’ consumption (more than once a week; score1) and ‘frequent’ consumption (at least once per day; score 2). We considered as “pro-Mediterranean” foods vegetables (raw green and cooked), fruit, fruit juice and fish, and as “anti-Mediterranean” foods meat, burger and fizzy drinks. Scores for the “anti- Mediterranean” foods were reversed (i.e. 2= low consumption, 0= frequent consumption). The points for each of the 8 items were added up to a summary score [5].

*Affluent centres*

Affluent centres are: Dresden, Munich (both Germany), Athens, Thessaloniki (both Greece), Rome (Italy), Utrecht (Netherlands), Hawkes Bay (New Zealand), Tromso (Norway), Almeria, Cartagena, Madrid, Valencia (all Spain), Linkoeping, Oestersund (both Sweden)

References

1. Weiland SK, Björkstén B, Brunekreef B, Cookson WOC, von Mutius E, Strachan DP: Phase II of the International Study of Asthma and Allergies in Childhood (ISAAC II): rationale and methods. The European Respiratory Journal : Official Journal of the European Society for Clinical Respiratory Physiology 2004;24:406-12.
2. Miller MR, Hankinson J, Brusasco V, Burgos F, Casaburi R, Coates A, et al: Standardisation of spirometry. European Respiratory Journal 2005;26(2):319–38.
3. Riedler J, Reade T, Dalton M, Holst D, Robertson C: Hypertonic saline challenge in an epidemiologic survey of asthma in children. American Journal of Respiratory and Critical Care Medicine 1994;150:1632–1639.
4. Saraçlar Y, Kuyucu S, Tuncer A, Sekerel B, Saçkesen C, Kocabaş C: Prevalence of asthmatic phenotypes and bronchial hyperresponsiveness in Turkish schoolchildren: an International Study of Asthma and Allergies in Childhood (ISAAC) phase 2 study. Annals of Allergy, Asthma & Immunology. 2003;91(5):477-84.
5. [Nagel G](http://www.ncbi.nlm.nih.gov/pubmed?term=Nagel G%5BAuthor%5D&cauthor=true&cauthor_uid=20522849), [Weinmayr G](http://www.ncbi.nlm.nih.gov/pubmed?term=Weinmayr G%5BAuthor%5D&cauthor=true&cauthor_uid=20522849), [Kleiner A](http://www.ncbi.nlm.nih.gov/pubmed?term=Kleiner A%5BAuthor%5D&cauthor=true&cauthor_uid=20522849), [Garcia-Marcos L](http://www.ncbi.nlm.nih.gov/pubmed?term=Garcia-Marcos L%5BAuthor%5D&cauthor=true&cauthor_uid=20522849), [Strachan DP](http://www.ncbi.nlm.nih.gov/pubmed?term=Strachan DP%5BAuthor%5D&cauthor=true&cauthor_uid=20522849); [ISAAC Phase Two Study Group](http://www.ncbi.nlm.nih.gov/pubmed?term=ISAAC Phase Two Study Group%5BCorporate Author%5D). Effect of diet on asthma and allergic sensitisation in the International Study on Allergies and Asthma in Childhood (ISAAC) Phase Two. Thorax 2010; 65(6):516-22.
