# Supplementary material for: Overweight/Obesity and Respiratory and Allergic Disease in Children: International Study of Asthma and Allergies in Childhood (ISAAC) Phase Two
Source: PLoS One. 2014 Dec 4;9(12):e113996. doi: 10.1371/journal.pone.0113996 (PMC4256390; doi:10.1371/journal.pone.0113996)
Supplement: File S2 — Additional Tables: Table S1: Mean and prevalence of investigated outcomes with 95% confidence interval. Table S2: Test for confounding: results from fully adjusted model including all potential factors tested. Table S3: Association of wheeze and related symptoms with overweight and obesity: combined estimates from meta-analysis, adjusted for sex. Table S4: Association of respiratory and allergy related outcomes and eczema with overweight and obesity, adjusted for sex. Table S5: Association of cough and phlegm with overweight and obesity: combined estimates from meta-analysis adjusted for sex. Table S6: Association of overweight and obesity with respiratory and allergy related outcomes and eczema in the absence/presence of wheeze: combined estimates with 95%-confidence intervals from meta-analysis adjusted for sex. Table S7: Association of respiratory and allergy related outcomes and eczema with overweight and obesity: analysis by sex. (DOC) [file pone.0113996.s002.doc]

**Table S1:** Mean and prevalence # of investigated outcomes with 95% confidence interval

|  | **Wheeze with exercise** | **Wheeze without exercise** | **Woken with tightness of chest** | **Dry cough at night** | **Woken with shortness of breath** | **Severe wheeze** | **BHR yes/no** | **Skin prick test** | **Total IgE &** | **rhinitis** |
| --- | --- | --- | --- | --- | --- | --- | --- | --- | --- | --- |
| Brasil, Uruguaiana$ | 14.4 (12.1;16.6) | 7.8 (6.1;9.5) | 24.7 (22.0;27.5) | 38.7 (35.6;41.8) | 25.5 (22.7;28.3) | 15.5 (13.1;18.0) | NA | 12.7 (10.6;14.8) | NA | 30.0 (27.1;32.9) |
| Estonia, Tallinn* | 12.6 (8.3;16.9) | 7.5 (4.0;10.9) | 6.3 (3.2;9.5) | 12.0 (7.9;16.2) | 8.0 (4.5;11.5) | 6.1 (2.7;9.4) | 3.6 (1.3;5.9) | 13.6 (8.4;18.8) | 44.7 (36.2;53.2) | 18.1 (13.1;23.0) |
| Georgia, Tbilisi* | 11.0 (6.0;15.9) | 6.5 (2.6;10.5) | 14.8 (9.2;20.5) | 21.8 (15.5;28.2) | 34.1 (26.8;41.5) | 12.2 (6.5;17.9) | 29.7 (22.1;37.3) | 33.5 (25.5;41.5) | 58.9 (50.5;67.3) | 57.6 (50.0;65.2) |
| Germany, Dresden§ | 5.4 (3.7;7.1) | NA | NA | 15.2 (12.5;17.9) | NA | 2.9 (1.6;4.2) | 10.7 (8.3;13.1) | 26.8 (23.4;30.2) | 43.2 (38.9;47.4) | 22.2 (19.1;25.4) |
| Germany, Munich§ | 6.5 (4.9;8.2) | NA | NA | 18.6 (16.0;21.2) | NA | 4.4 (3.0;5.7) | 17.9 (15.2;20.7) | 21.7 (18.8;24.5) | 44 (40.0;48.0) | 19.1 (16.5;21.7) |
| Ghana, Kintampo* | 25.3 (19.8;30.8) | 16.6 (11.9;21.3) | 6.2 (3.2;9.3) | 63.1 (56.9;69.2) | 14.5 (10.0;19.0) | 22.0 (16.2;27.7) | 29.8 (22.8;36.7) | 1.7 (0;3.3) | NA | 25.0 (19.5;30.5) |
| Greece, Athens* | 7.3 (3.6;10.9) | 8.8 (4.8;12.8) | 4.7 (1.7;7.7) | 28.0 (21.6;34.4) | 15.5 (10.4;20.7) | 7.1 (3.2;11.1) | 13.6 (8.3;18.8) | 29.5 (23.0;36.0) | 54.1 (46.0;62.3) | 27.5 (21.1;33.8) |
| Greece, Thessaloniki* | 11.4 (7.1;15.7) | 15.6 (10.7;20.6) | 8.6 (4.8;12.4) | 32.4 (26.0;38.8) | 22.4 (16.7;28.1) | 13.2 (7.9;18.5) | 39.9 (32.2;47.7) | 34.1 (27.7;40.6) | 47.6 (39.8;55.5) | 35.2 (28.7;41.8) |
| India, Mumbai* | 8.8 (3.5;14.0) | 3.5 (0.1;6.9) | 4.4 (0.6;8.2) | 16.9 (10.1;23.8) | 8.8 (3.5;14.0) | 10.4 (4.2;16.6) | 47.6 (35.9;59.3) | 10.4 (4.8;16.1) | 90.8 (83.8;97.7) | 13.4 (7.2;19.7) |
| Italy, Rome$ | 37.4 (27.7;47.1) | 51.5 (41.5;61.5) | 9.7 (8.1;11.3) | 19.0 (16.8;21.2) | 16.7 (14.6;18.7) | 2.3 (1.4;3.1) | 36.8 (27.9;45.6) | 28.9 (26.4;31.4) | 55.7 (43.7;67.7) | 21.4 (19.1;23.6) |
| Latvia, Riga§ | 8.4 (4.0;12.8) | 6.5 (2.6;10.4) | 5.3 (1.7;8.9) | 13.7 (8.2;19.2) | 15.5 (9.7;21.2) | 0.7 (-0.7;2.1) | 12.4 (7.1;17.7) | 18.3 (11.9;24.7) | NA | 33.1 (25.6;40.6) |
| Netherlands, Utrecht$ | 7.3 (6.3;8.3) | 5.6 (4.7;6.4) | NA | 21.7 (20.1;23.3) | NA | 4.1 (3.3;4.9) | 19.8 (17.4;22.3) | 30.6 (28.0;33.2) | 47.7 (45.2;50.2) | 25.0 (23.3;26.6) |
| New Zealand, Hawkes Bay* | 44.6 (38.0;51.2) | NA | NA | 39.4 (32.9;45.9) | NA | 36.9 (29.7;44.1) | 23.7 (17.7;29.7) | 36.9 (30.5;43.3) | 49.5 (41.9;57.1) | 45.9 (39.3;52.6) |
| Norway, Tromso* | 42.1 (34.4;49.7) | 42.3 (34.7;50.0) | 44.4 (36.6;52.2) | 19.3 (16.1;22.4) | 29.6 (22.5;36.7) | 11.3 (8.7;13.9) | 42.8 (31.1;54.4) | 32.1 (28.3;35.9) | 33.3 (24.1;42.5) | 23.0 (19.7;26.3) |
| Palestine, Ramallah* | NA | NA | NA | 26.1 (20.1;32.0) | NA | 10.4 (6.0;14.7) | NA | 10.4 (6.2;14.5) | NA | 27.5 (21.4;33.6) |
| Spain, Almeria* | 27.9 (21.6;34.1) | 18.6 (13.1;24.0) | 19.5 (14.0;25.0) | 39.5 (32.8;46.3) | 27.7 (21.5;33.9) | 24.8 (17.5;32.1) | 29.7 (22.3;37.2) | 52.0 (45.0;59.0) | 58.9 (48.2;69.6) | 47.6 (40.7;54.4) |
| Spain, Cartagena* | 17.5 (11.5;23.6) | 21.2 (14.6;27.8) | 16.1 (10.3;22.0) | 33.3 (25.9;40.7) | 26.1 (19.2;33.1) | 22.4 (14.7;30.1) | 23.7 (16.5;30.9) | 37.0 (28.8;45.3) | 41.2 (14.4;68.1) | 37.3 (29.5;45.0) |
| Spain, Madrid* | 14.7 (11.2;18.2) | 10.5 (7.4;13.5) | 16.6 (12.9;20.3) | 33.3 (28.6;37.9) | 24.6 (20.4;28.9) | 10.1 (7.0;13.2) | 8.9 (6.4;11.5) | 37.7 (32.9;42.4) | 51.8 (46.2;57.3) | 39.1 (34.2;43.9) |
| Spain, Valencia* | 8.0 (4.1;12.0) | 7.4 (3.7;11.2) | 10.7 (6.2;15.2) | 29.8 (23.2;36.4) | 16.7 (11.3;22.1) | 3.0 (0.4;5.5) | 24.4 (17.3;31.5) | 16.5 (10.9;22.0) | 54.3 (44.0;64.7) | 39.5 (32.5;46.5) |
| Sweden, Linkoeping* | NA | NA | NA | 19.1 (13.3;24.9) | NA | 14.1 (8.3;19.9) | 19.1 (12.5;25.7) | 29.5 (22.5;36.5) | 43.1 (34.5;51.8) | 30.9 (23.9;37.8) |
| Sweden, Oestersund* | NA | NA | NA | 20.9 (16.0;25.7) | NA | 23.9 (18.2;29.5) | 33.8 (26.2;41.3) | 42.3 (36.0;48.6) | 43.3 (36.4;50.2) | 36.8 (31.1;42.5) |
| Turkey, Ankara* | 20.3 (16.0;24.6) | 12.7 (9.1;16.3) | 17.1 (13.0;21.2) | 43.2 (37.9;48.6) | 20.3 (16.0;24.6) | 20.5 (15.3;25.8) | 22.5 (16.9;28.0) | 24.9 (20.3;29.5) | NA | 41.8 (36.5;47.1) |

|  | **Eczema past year** | **Eczema by examination** | **FEV1/FVC (%)** | **FEV1 (ml)** | **FVC (ml)** | **Coughed up phlegm with colds** | **Coughed up phlegm without colds** | **Congested in chest/coughed up phlegm frequently** |
| --- | --- | --- | --- | --- | --- | --- | --- | --- |
| Brasil, Uruguaiana$ | 14.4 (12.1;16.6) | NA | NA | NA | NA | 55.6 (52.4;58.7) | 12.4 (10.3;14.5) | 14.9 (12.6;17.2) |
| Estonia, Tallinn* | 13.9 (9.4;18.3) | 3.6 (0.7;6.4) | NA | 2809.39 (2747.55;2871.23) | NA | 43.5 (37.1;49.8) | 6.4 (3.3;9.6) | 4.5 (1.8;7.3) |
| Georgia, Tbilisi* | 22.2 (15.6;28.7) | 14.2 (8.3;20.1) | 96.7 (96.0;97.5) | 2194.13 (2114.82;2273.44) | 2264.47 (2178.13; 2350.81) | 68.6 (61.6;75.7) | 21.4 (14.9;28.0) | 20.7 (13.9;27.5) |
| Germany, Dresden§ | 15.8 (13.1;18.5) | 5.3 (3.7;7.0) | 91.2 (90.8;91.7) | 2203.66 (2178.43;2228.89) | 2423.69 (2394.53; 2452.84) | NA | NA | NA |
| Germany, Munich§ | 9.8 (7.8;11.8) | 4.3 (3.0;5.6) | 89.4 (89.0;89.8) | 2133.19 (2112.62;2153.77) | 2394.73 (2370.52;2418.94) | NA | NA | NA |
| Ghana, Kintampo* | 5.8 (2.8;8.8) | 0.4 (-0.4;1.2) | NA | 1718.73 (1676.12;1761.35) | NA | 38.6 (32.4;44.8) | 17.0 (12.2;21.8) | 3.3 (0.9;5.8) |
| Greece, Athens* | 10.4 (6.0;14.7) | 1.6 (-0.2;3.3) | NA | 2227.03 (2173.69;2280.38) | NA | 42.5 (35.5;49.5) | 10.9 (6.4;15.3) | 6.5 (2.9;10.1) |
| Greece, Thessaloniki* | 4.8 (1.9;7.7) | 1.4 (-0.2;3.0) | NA | 2216.46 (2153.99;2278.94) | NA | 51.0 (44.1;57.8) | 16.6 (11.5;21.6) | 9.8 (5.6;14.0) |
| India, Mumbai* | 7.6 (2.7;12.4) | 0.8 (-0.8;2.5) | NA | 1499.20 (1425.65;1572.75) | NA | 22.7 (15.1;30.3) | 13.4 (7.2;19.7) | 10.2 (4.6;15.7) |
| Italy, Rome$ | 6.8 (5.4;8.1) | 1.6 (0.9;2.3) | NA | 2290.92 (2225.82;2356.03) | NA | 55.7 (53.0;58.4) | 10.3 (8.6;12.0) | 4.4 (3.3;5.6) |
| Latvia, Riga§ | 19.4 (13.1;25.6) | 5.4 (0.7;10.0) | NA | 2917.92 (2846.52;2989.31) | NA | 49.7 (41.7;57.6) | 11.2 (6.1;16.3) | 2.2 (-0.3;4.8) |
| Netherlands, Utrecht$ | 12.3 (11.1;13.6) | 5.4 (4.1;6.7) | 88.6 (88.4;88.9) | 2219.68 (2203.46;2235.90) | 2510.79 (2491.57; 2530.00) | 32.7 (30.9;34.5) | 9.5 (8.4;10.6) | 3.6 (2.9;4.4) |
| New Zealand, Hawkes Bay* | 15.3 (10.5;20.1) | 7.7 (4.1;11.2) | 89.9 (88.7;91.1) | 2344.14 (2288.50;2399.78) | 2622.97 (2551.60; 2694.33) | NA | NA | NA |
| Norway, Tromso* | 18.5 (15.5;21.6) | 9.7 (7.2;12.2) | 89.2 (88.7;89.8) | 2318.71 (2289.01;2348.41) | 2592.21 (2536.40; 2648.02) | 23.3 (19.9;26.7) | 7.5 (5.4;9.6) | 2.5 (1.3;3.8) |
| Palestine, Ramallah* | 12.9 (8.3;17.4) | 2.9 (0.5;5.2) | NA | 1561.96 (1490.20;1633.72) | NA | NA | NA | NA |
| Spain, Almeria* | 10.8 (6.5;15.2) | 0 | 91.7 (90.6;92.9) | 2447.31 (2379.93;2514.69) | 2661.24 (2582.57; 2739.90) | 63.6 (57.0;70.2) | 22.2 (16.4;27.9) | 21.1 (14.9;27.2) |
| Spain, Cartagena* | 7.9 (3.6;12.2) | 0.7 (-0.6;2.0) | 91.3 (90.2;92.3) | 2036.88 (1972.42;2101.34) | 2242.79 (2163.52; 2322.05) | 65.8 (58.3;73.3) | 18.7 (12.5;24.9) | 15.0 (9.0;21.0) |
| Spain, Madrid* | 13.4 (10.0;16.8) | 3.3 (1.5;5.0) | NA | 2001.71 (1971.09;2032.33) | NA | 63.9 (59.2;68.6) | 17.7 (13.9;21.5) | 18.0 (14.0;22.0) |
| Spain, Valencia* | 11.4 (6.8;16.1) | 6.5 (2.9;10.0) | NA | 1993.86 (1940.56;2047.17) | NA | 50.3 (43.1;57.5) | 13.8 (8.8;18.7) | 8.6 (4.4;12.8) |
| Sweden, Linkoeping* | 28.8 (22.1;35.6) | 8.2 (3.8;12.6) | NA | 2752.47 (2672.21;2832.73) | NA | 34.4 (27.4;41.5) | 9.6 (5.2;13.9) | 3.5 (0.7;6.3) |
| Sweden, Oestersund* | 30.5 (25.1;36.0) | 14.2 (9.6;18.9) | NA | 2536.59 (2475.37;2597.81) | NA | 39.2 (33.4;45.0) | 11.3 (7.5;15.0) | 3.1 (1.0;5.2) |
| Turkey, Ankara* | 8.3 (5.3;11.3) | 1.8 (0.4;3.2) | 86.2 (85.2;87.1) | 1937.16 (1898.01;1976.31) | 2267.05 (2210.64; 2323.45) | 51.8 (46.4;57.1) | 25.1 (20.5;29.8) | 19.8 (15.4;24.2) |

*: Stratified random subsample; §: Random sample; $ Full sample
# Estimates referring to the full sample mean and prevalence are reported (implying appropriate weighting for stratified subsamples), & dichotomized at 4.17 kU/l

**Table S2:** Test for confounding: results from fully adjusted model including all potential factors* tested

|  |  | Overweight |  |  |  | Obesity |  |  |
| --- | --- | --- | --- | --- | --- | --- | --- | --- |
|  |  | OR (95%-CI) | N | N centres |  | OR (95%-CI) | N | N centres |
| **Wheeze** |  |  |  |  |  |  |  |  |
| Crude OR |  | 1.14 (0.98;1.33) | 9658 | 21 |  | 1.67 (1.25;2.21) | 7274 | 18 |
| Adjusted OR |  | 1.14 (0.92;1.41) | 4200 | 15 |  | 1.67 (1.19;2.33) | 3187 | 13 |
| Crude in reduced data set | | 1.15 (0.95;1.40) | 4200 | 15 |  | 1.64 (1.13;2.37) | 3187 | 13 |
| **BHR** |  |  |  |  |  |  |  |  |
| Crude OR |  | 1.05 (0.88;1.26) | 5056 | 19 |  | 1.09 (0.81;1.47) | 3916 | 16 |
| Adjusted OR |  | 0.96 (0.69;1.33) | 1743 | 13 |  | 0.97 (0.62;1.52) | 1304 | 11 |
| Crude in reduced data set | | 0.94 (0.69;1.27) | 1743 | 13 |  | 0.95 (0.63;1.42) | 1304 | 11 |
| **FEV1/FVC $** |  |  |  |  |  |  |  |  |
| Crude mean increase (%) | | -0.90 (-1.33;-0.47) | 5439 | 9 |  | -2.46 (-3.84;-1.07) | 4746 | 9 |
| Adjusted mean increase (%) | | -0.52 (-1.61;0.57) | 1185 | 6 |  | -2.49 (-4.58;-0.41) | 1033 | 6 |
| Crude in reduced data set | | -0.59 (-1.65;0.46) | 1185 | 6 |  | -2.48 (-4.73;-0.23) | 1033 | 6 |
| **Skin prick test** | |  |  |  |  |  |  |  |
| Crude OR |  | 1.04 (0.91;1.18) | 7891 | 21 |  | 1.13 (0.91;1.42) | 6194 | 18 |
| Adjusted OR |  | 0.94 (0.79;1.13) | 3981 | 14 |  | 1.24 (0.93;1.66) | 3088 | 13 |
| Crude in reduced data set | | 0.93 (0.78;1.10) | 3981 | 14 |  | 1.26 (0.95;1.67) | 3088 | 13 |
| **Total IgE (cutpoint: 4.17kU/l)** | | |  |  |  |  |  |  |
| Crude OR |  | 0.95 (0.80;1.12) | 4451 | 15 |  | 1.07 (0.82;1.39) | 3709 | 15 |
| Adjusted OR |  | 0.80 (0.58;1.10) | 1123 | 9 |  | 1.22 (0.77;1.95) | 920 | 9 |
| Crude in reduced data set | | 0.79 (0.59;1.05) | 1123 | 9 |  | 1.41 (0.79;2.50) | 920 | 9 |
| **Rhinitis** |  |  |  |  |  |  |  |  |
| Crude OR |  | 0.99 (0.88;1.11) | 9522 | 21 |  | 1.19 (0.90;1.56) | 7746 | 19 |
| Adjusted OR |  | 0.91 (0.76;1.07) | 4153 | 15 |  | 1.22 (0.86;1.74) | 3147 | 13 |
| Crude in reduced data set | | 0.97 (0.82;1.13) | 4153 | 15 |  | 1.24 (0.89;1.72) | 3147 | 13 |
| **Eczema past year** | |  |  |  |  |  |  |  |
| Crude OR |  | 1.19 (1.02;1.39) | 9376 | 20 |  | 1.45 (0.94;2.24) | 7471 | 18 |
| Adjusted OR |  | 1.21 (0.93;1.59) | 4061 | 14 |  | 1.33 (0.71;2.50) | 2824 | 11 |
| Crude in reduced data set | | 1.20 (0.96;1.50) | 4061 | 14 |  | 1.27 (0.71;2.27) | 2824 | 11 |
| **Eczema by examination** | |  |  |  |  |  |  |  |
| Crude OR |  | 1.35 (0.98;1.85) | 6348 | 15 |  | 1.18 (0.70;2.00) | 4292 | 11 |
| Adjusted OR |  | 1.34 (0.83;2.18) | 2300 | 7 |  | 2.00 (0.92;4.32) | 1698 | 6 |
| Crude in reduced data set | | 1.36 (0.85;2.18) | 2300 | 7 |  | 1.85 (0.90;3.78) | 1698 | 6 |
| **Coughed up phlegm with colds** | | |  |  |  |  |  |  |
| Crude OR |  | 1.11 (0.94;1.30) | 7646 | 17 |  | 1.26 (0.96;1.66) | 6251 | 16 |
| Adjusted OR |  | 1.09 (0.93;1.27) | 3795 | 13 |  | 1.35 (1.07;1.71) | 2947 | 12 |
| Crude in reduced data set | | 1.09 (0.94;1.27) | 3795 | 13 |  | 1.35 (1.08;1.69) | 2947 | 12 |
| **Coughed up phlegm without colds** | | |  |  |  |  |  |  |
| Crude OR |  | 1.17 (0.98;1.40) | 7541 | 17 |  | 1.64 (1.15;2.34) | 5840 | 14 |
| Adjusted OR |  | 1.18 (0.92;1.51) | 3738 | 13 |  | 1.80 (1.29;2.52) | 2783 | 11 |
| Crude in reduced data set | | 1.18 (0.93;1.48) | 3738 | 13 |  | 1.81 (1.33;2.48) | 2783 | 11 |
|  |  |  |  |  |  |  |  |  |

* Sex, age, parental allergy, smoking pregnancy, birthweight, dampness, ETS, maternal education, breastfeeding

$ Adjusted for height in addition

OR: Odds ratios

**Table S3:** Association of wheeze and related symptoms with overweight and obesity: combined estimates from meta-analysis, adjusted for sex

|  | **Overweight** |  |  | **Obese** |  |  |
| --- | --- | --- | --- | --- | --- | --- |
|  | **OR (95%-CI)** | **N** | **n*** | **OR (95%-CI)** | **N** | **n*** |
| **Wheeze past year** |  |  |  |  |  |  |
| N-C | 1.31 (0.95;1.80) | 5095 | 6 | 2.84 (1.67;4.82) | 3838 | 5 |
| S | 1.19 (0.93;1.51) | 2331 | 7 | 1.31 (0.91;1.89) | 1861 | 7 |
| **Wheeze with exercise** |  |  |  |  |  |  |
| N-C | 1.22 (0.90;1.65) | 4167 | 4 | 1.46 (0.54;3.97) | 3629 | 4 |
| S | 1.40 (0.86;2.28) | 1216 | 7 | 1.29 (0.80;2.06) | 1002 | 7 |
| **Wheeze without exercise** | |  |  |  |  |  |
| N-C | 0.94 (0.62;1.44) | 2662 | 2 | 0.92 (0.35;2.48) | 2333 | 2 |
| S | 1.26 (0.70;2.27) | 1208 | 7 | 1.16 (0.71;1.92) | 996 | 7 |
| **Sleep disturbing wheeze** | |  |  |  |  |  |
| N-C | 1.79 (0.87;3.70) | 4814 | 5 | 2.99 (1.13;7.90) | 3684 | 4 |
| S | 0.75 (0.30;1.88) | 970 | 5 | 1.39 (0.54;3.58) | 1712 | 6 |
| **Dry cough at night** |  |  |  |  |  |  |
| N-C | 1.28 (1.07;1.54) | 5021 | 6 | 1.64 (1.19;2.25) | 4366 | 6 |
| S | 1.16 (0.93;1.44) | 2269 | 7 | 1.08 (0.80;1.45) | 1806 | 7 |
| **Woken with shortness of breath** | |  |  |  |  |  |
| N-C | Only one centre left |  |  | Only one centre left |  |  |
| S | 1.11 (0.85;1.45) | 2269 | 7 | 1.41 (0.82;2.43) | 1687 | 6 |
| **Severe wheeze** |  |  |  |  |  |  |
| N-C | 1.36 (0.92;2.03) | 4758 | 6 | 2.54 (1.28;5.05) | 3572 | 5 |
| S | 1.28 (0.74;2.22) | 2052 | 7 | 1.63 (0.86;3.06) | 1633 | 7 |
| **Severe wheeze among wheezers** | |  |  |  |  |  |
| N-C | 0.94 (0.63;1.42) | 603 | 6 | 0.84 (0.39;1.79) | 482 | 5 |
| S | 1.20 (0.74;1.94) | 368 | 6 | 1.37 (0.77;2.44) | 313 | 6 |

* Number of centres
N-C: Germany, The Netherlands, Norway, Sweden
S: Greece, Italy, Spain

OR: Odds ratios

**Table S4**: Association of respiratory and allergy related outcomes and eczema with overweight and obesity, adjusted for sex

|  | **Overweight** | | | | **Obese** | | | |
| --- | --- | --- | --- | --- | --- | --- | --- | --- |
|  | OR (95%-CI) | | N | n* | OR (95%-CI) | | N | n* |
| **BHR yes/no** | | | | |  | |  |  |
| N-C | 1.23 (0.95;1.58) | | 2776 | 6 | 1.56 (0.96;2.51) | | 2387 | 6 |
| S | 1.02 (0.71;1.45) | | 1175 | 7 | 0.88 (0.56;1.36) | | 970 | 7 |
| **Skin prick test** | | | | |  | |  |  |
| N-C | 1.11 (0.92;1.36) | | 3518 | 6 | 1.16 (0.78;1.73) | | 3036 | 6 |
| S | 0.97 (0.79;1.19) | | 2239 | 7 | 1.09 (0.83;1.43) | | 1798 | 7 |
| **Total IgE $** | | | | |  | |  |  |
| N-C | 1.07 (0.88;1.30) | | 3093 | 6 | 1.73 (0.76;3.97) | | 2656 | 6 |
| S | 0.84 (0.62;1.15) | | 859 | 6 | 0.86 (0.57;1.30) | | 751 | 7 |
| **Rhinitis** | | | | |  | |  |  |
| N-C | 0.93 (0.77;1.14) | | 5021 | 6 | 1.19 (0.73;1.95) | | 4367 | 6 |
| S | 0.99 (0.81;1.22) | | 2288 | 7 | 0.99 (0.66;1.50) | | 1821 | 7 |
| **Rhinitis without wheeze** | | | | |  | |  |  |
| N-C | 0.83 (0.60;1.17) | | 4419 | 6 | 1.21 (0.81;1.81) | | 3700 | 5 |
| S | 1.01 (0.80;1.28) | | 1887 | 7 | 0.99 (0.61;1.60) | | 1487 | 7 |
| **Reported eczema past year** | | | | |  | |  |  |
| N-C | 1.19 (0.96;1.48) | | 5027 | 6 | 2.30 (1.05;5.04) | | 4377 | 6 |
| S | 1.43 (1.04;1.97) | | 2270 | 7 | 1.12 (0.48;2.60) | | 1689 | 6 |
| **Reported eczema without wheeze** | | | | |  | |  |  |
| N-C | 1.21 (0.95;1.54) | | 4428 | 6 | 1.90 (0.93;3.91) | | 3850 | 6 |
| S | 1.38 (0.95;2.01) | | 1760 | 6 | 1.03 (0.58;1.82) | | 1323 | 5 |
| **Eczema by examination** | | | |  |  | |  |  |
| N-C | 1.33 (0.92;1.92) | | 3628 | 6 | 1.06 (0.50;2.24) | | 2533 | 5 |
| S | 1.31 (0.43;3.98) | | 1680 | 3 | 1.20 (0.48;3.00) | | 1451 | 4 |
| **Examined eczema without wheeze** | | | | |  | |  |  |
| N-C | 1.30 (0.77;2.19) | | 3199 | 6 | 1.86 (0.62;5.58) | | 1407 | 3 |
| S | 1.42 (0.31;6.53) | | 1501 | 3 | 2.52 (0.85;7.46) | | 1071 | 2 |
| **FEV1/FVC &#** | mean change (%) (95%-CI) | | N | n* | mean change (%)  (95%-CI) | | N | n* |
| N-C | -1.04 (-1.61;-0.47) | | 4476 | 4 | -1.71 (-2.49;-0.93) | | 3901 | 4 |
| S | -1.09 (-2.91;0.74) | | 303 | 2 | -0.53 (-2.77;1.71) | | 257 | 2 |
| **FEV1 &** | | mean change (ml) |  | | | mean change (ml) |  |  |
| N-C | 72.04 (52.91;91.17) | | 5036 | 6 | 81.32 (44.34;118.31) | | 4379 | 6 |
| S | 34.24 (1.96;66.53) | | 1265 | 7 | 97.28 (36.17;158.39) | | 1037 | 7 |
| **FVC &** | | mean change (ml) |  | | | mean change (ml) |  |  |
| N-C | 109.84 (87.09;132.58) | | 4524 | 4 | 158.00 (109.08;206.92) | | 3940 | 4 |
| S | 52.66 (-37.71;143.04) | | 305 | 2 | 217.45 (99.73;335.18) | | 259 | 2 |

* Number of centres
N-C: Germany, The Netherlands, Norway, Sweden;
S: Greece, Italy, Spain

$ Dichotomized at 4.17 kU/l
& Adjusted for age, sex and height
# Exclusion of children with difference of FEV1 and FVC>200 ml or difference=-12 000 ml

OR: Odds ratios

**Table S5**: Association of cough and phlegm with overweight and obesity: combined estimates from meta-analysis adjusted for sex

|  | **Overweight** | | | **Obese** | | |
| --- | --- | --- | --- | --- | --- | --- |
|  | **OR (95%-CI)** | **N** | **n*** | **OR (95%-CI)** | **N** | **n*** |
| **Coughed up phlegm with colds** | | | |  |  |  |
| N-C | 1.40 (1.10;1.78) | 3525 | 4 | 2.06 (1.46;2.91) | 3075 | 4 |
| S | 1.10 (0.88;1.37) | 2299 | 7 | 1.03 (0.67;1.58) | 1833 | 7 |
| **Coughed up phlegm with colds without wheeze** | | | |  |  |  |
| N-C | 0.99 (0.54;1.84) | 3052 | 4 | 1.62 (1.04;2.50) | 2660 | 4 |
| S | 1.08 (0.84;1.39) | 1894 | 7 | 1.08 (0.72;1.63) | 1498 | 7 |
| **Coughed up phlegm without colds** | | | |  |  |  |
| N-C | 1.49 (0.79;2.81) | 3505 | 4 | 2.51 (1.61;3.90) | 3058 | 4 |
| S | 1.24 (0.89;1.74) | 2250 | 7 | 1.34 (0.69;2.63) | 1801 | 7 |
| **Coughed up phlegm without colds without wheeze** | | | |  |  |  |
| N-C | 1.11 (0.72;1.71) | 2405 | 2 | 2.15 (1.20;3.84) | 2552 | 3 |
| S | 1.30 (0.70;2.41) | 1771 | 6 | 1.99 (0.84;4.71) | 1294 | 5 |
| **Congested in chest/coughed up phlegm frequently§** | | | |  |  |  |
| N-C | 1.75 (1.10;2.78) | 3193 | 3 | 3.02 (1.52;6.01) | 2932 | 4 |
| S | 1.68 (1.18;2.39) | 2170 | 7 | 2.26 (1.22;4.21) | 1614 | 6 |
| **Congested in chest/coughed up phlegm frequently§ without wheeze** | | | | |  |  |
| N-C | Only one centre left | | | 6.36 (0.98;41.38) | 2014 | 2 |
| S | 1.30 (0.70;2.41) | 1727 | 6 | 1.99 (0.84;4.71) | 1256 | 5 |

* Number of centres
N-C: Germany, The Netherlands, Norway, Sweden;
S: Greece, Italy, Spain

§ On 4 or more days a week for as much as 3 months a year

OR: Odds ratios

**Table S6:** Association of overweight and obesity with respiratory and allergy related outcomes and eczema in the absence/presence of wheeze: combined estimates with 95%-confidence intervals from meta-analysis adjusted for sex

|  | **Overweight** |  |  | **Obese** |  |  |
| --- | --- | --- | --- | --- | --- | --- |
|  | OR (95%-CI) | N | n* | OR (95%-CI) | N | n* |
| **Rhinitis with wheeze** |  |  |  |  |  |  |
| All centres | 1.10 (0.93;1.32) | 7161 | 21 | 1.73 (1.26;2.38) | 5379 | 18 |
| Affluent | 1.12 (0.91;1.38) | 5752 | 14 | 1.65 (1.10;2.46) | 4426 | 13 |
| N-C | 1.22 (0.91;1.63) | 3921 | 6 | 2.99 (1.91;4.69) | 2961 | 5 |
| S | 1.07 (0.75;1.52) | 1697 | 7 | 1.12 (0.73;1.73) | 1354 | 7 |
| Nonaffluent | 1.06 (0.76;1.48) | 1409 | 7 | 1.99 (1.24;3.19) | 953 | 5 |
| **Rhinitis without wheeze** | |  |  |  |  |  |
| All centres | 0.99 (0.86;1.13) | 7878 | 21 | 1.13 (0.85;1.51) | 6243 | 18 |
| Affluent | 0.96 (0.83;1.12) | 6408 | 14 | 1.03 (0.75;1.42) | 5274 | 13 |
| N-C | 0.83 (0.60;1.17) | 4419 | 6 | 1.21 (0.81;1.81) | 3700 | 5 |
| S | 1.01 (0.80;1.28) | 1887 | 7 | 0.99 (0.61;1.60) | 1487 | 7 |
| Nonaffluent | 1.07 (0.80;1.43) | 1470 | 7 | 1.56 (0.79;3.08) | 969 | 5 |
| **Reported eczema with wheeze** | |  |  |  |  |  |
| All centres | 1.33 (1.02;1.75) | 7025 | 18 | 2.82 (1.59;5.00) | 4555 | 15 |
| Affluent | 1.48 (1.08;2.02) | 5874 | 13 | 3.36 (1.60;7.09) | 3677 | 10 |
| N-C | 1.47 (0.94;2.32) | 4093 | 6 | 5.64 (1.99;16.03) | 3094 | 5 |
| S | 1.49 (0.82;2.72) | 1671 | 6 | 1.75 (0.57;5.31) | 489 | 4 |
| Nonaffluent | 0.83 (0.39;1.74) | 1151 | 5 | 1.62 (0.82;3.21) | 878 | 5 |
| **Reported eczema without wheeze** | |  |  |  |  |  |
| All centres | 1.18 (0.99;1.41) | 7667 | 19 | 1.24 (0.88;1.75) | 5900 | 15 |
| Affluent | 1.26 (1.03;1.54) | 6290 | 13 | 1.34 (0.93;1.92) | 5260 | 12 |
| N-C | 1.21 (0.95;1.54) | 4428 | 6 | 1.90 (0.93;3.91) | 3850 | 6 |
| S | 1.38 (0.95;2.01) | 1760 | 6 | 1.03 (0.58;1.82) | 1323 | 5 |
| Nonaffluent | 0.94 (0.64;1.37) | 1377 | 6 | 1.01 (0.35;2.94) | 640 | 3 |
| **Examined eczema with wheeze** | |  |  |  |  |  |
| All centres | 1.77 (1.04;3.00) | 2968 | 8 | 3.24 (1.41;7.48) | 2115 | 8 |
| Affluent | 1.85 (1.07;3.21) | 2867 | 7 | 3.39 (1.39;8.24) | 2031 | 7 |
| N-C | 1.88 (1.01;3.53) | 2486 | 5 | 3.35 (1.24;9.04) | 1756 | 4 |
| S | Only one centre left |  |  | 5.64 (0.32;98.90) | 186 | 2 |
| Nonaffluent | Only one centre left |  |  | Only one centre left |  |  |
| **Examined eczema without wheeze** | |  |  |  |  |  |
| All centres | 1.27 (0.88;1.82) | 5366 | 14 | 2.07 (1.03;4.17) | 2656 | 7 |
| Affluent | 1.29 (0.85;1.96) | 4802 | 10 | 2.25 (1.08;4.68) | 2565 | 6 |
| N-C | 1.30 (0.77;2.19) | 3199 | 6 | 1.86 (0.62;5.58) | 1407 | 3 |
| S | 1.42 (0.31;6.53) | 1501 | 3 | 2.52 (0.85;7.46) | 1071 | 2 |
| Nonaffluent | 1.15 (0.39;3.40) | 564 | 4 | Only one centre left |  |  |
| **Coughed up phlegm with colds with wheeze** | |  |  |  |  |  |
| All centres | 1.24 (1.00;1.55) | 5106 | 17 | 1.80 (1.21;2.68) | 4129 | 15 |
| Affluent | 1.49 (1.20;1.85) | 3890 | 11 | 2.07 (1.26;3.39) | 3322 | 11 |
| N-C | 1.85 (1.38;2.48) | 2601 | 4 | 4.17 (2.64;6.59) | 2282 | 4 |
| S | 1.21 (0.90;1.62) | 1289 | 7 | 1.35 (0.79;2.31) | 1040 | 7 |
| Nonaffluent | 0.89 (0.63;1.24) | 1216 | 6 | 1.37 (0.83;2.26) | 807 | 4 |
| **Coughed up phlegm with colds without wheeze** | |  |  |  |  |  |
| All centres | 1.03 (0.84;1.26) | 6255 | 17 | 1.25 (0.98;1.59) | 4949 | 15 |
| Affluent | 1.06 (0.82;1.38) | 4946 | 11 | 1.27 (0.95;1.70) | 4158 | 11 |
| N-C | 0.99 (0.54;1.84) | 3052 | 4 | 1.62 (1.04;2.50) | 2660 | 4 |
| S | 1.08 (0.84;1.39) | 1894 | 7 | 1.08 (0.72;1.63) | 1498 | 7 |
| Nonaffluent | 0.94 (0.72;1.22) | 1309 | 6 | 1.11 (0.71;1.74) | 791 | 4 |
| **Coughed up phlegm without colds with wheeze** | |  |  |  |  |  |
| All centres | 1.38 (1.10;1.74) | 5955 | 15 | 1.85 (1.27;2.71) | 4817 | 14 |
| Affluent | 1.53 (1.14;2.04) | 4880 | 11 | 2.06 (1.28;3.32) | 4095 | 11 |
| N-C | 1.61 (0.89;2.92) | 3020 | 4 | 3.46 (1.88;6.36) | 2628 | 4 |
| S | 1.51 (1.04;2.18) | 1860 | 7 | 1.48 (0.79;2.75) | 1467 | 7 |
| Nonaffluent | 1.03 (0.57;1.83) | 1075 | 4 | 1.40 (0.70;2.81) | 722 | 3 |
| **Coughed up phlegm without colds without wheeze** | | |  |  |  |  |
| All centres | 1.07 (0.83;1.37) | 5375 | 13 | 1.86 (1.32;2.63) | 4418 | 10 |
| Affluent | 1.10 (0.78;1.56) | 4176 | 8 | 1.88 (1.27;2.80) | 3846 | 8 |
| N-C | 1.11 (0.72;1.71) | 2405 | 2 | 2.15 (1.20;3.84) | 2552 | 3 |
| S | 1.30 (0.70;2.41) | 1771 | 6 | 1.99 (0.84;4.71) | 1294 | 5 |
| Nonaffluent | 1.13 (0.63;2.05) | 1199 | 5 | 1.78 (0.76;4.17) | 572 | 2 |
| **Congested in chest/coughed up phlegm frequently^$^ with wheeze** | | | | |  |  |
| All centres | 1.43 (1.09;1.87) | 5668 | 14 | 2.10 (1.22;3.62) | 4519 | 12 |
| Affluent | 1.64 (1.14;2.37) | 4628 | 10 | 2.34 (1.10;5.00) | 3825 | 9 |
| N-C | 1.85 (0.98;3.49) | 2808 | 3 | 3.93 (0.74;20.83) | 2463 | 3 |
| S | 1.55 (0.98;2.43) | 1820 | 7 | 1.92 (0.81;4.54) | 1362 | 6 |
| Nonaffluent | 1.12 (0.58;2.16) | 1040 | 4 | 1.89 (0.96;3.71) | 694 | 3 |
| **Congested in chest/coughed up phlegm frequently^$^ without wheeze** | | | | |  |  |
| All centres | 1.52 (1.00;2.30) | 4947 | 11 | 2.71 (1.76;4.17) | 3824 | 9 |
| Affluent | 1.87 (1.24;2.82) | 3915 | 7 | 2.93 (1.55;5.52) | 3270 | 7 |
| N-C | Only one centre left |  |  | 6.36 (0.98;41.38) | 2014 | 2 |
| S | 1.30 (0.70;2.41) | 1727 | 6 | 1.99 (0.84;4.71) | 1256 | 5 |
| Nonaffluent | 0.85 (0.46;1.60) | 1032 | 4 | 2.65 (1.28;5.50) | 554 | 2 |

* Number of centres; N-C: Germany, The Netherlands, Norway, Sweden; S: Greece, Italy, Spain;

$ On 4 or more days a week for as much as 3 months a year

OR: Odds ratios

**Table S7:** Association of respiratory and allergy related outcomes and eczema with overweight and obesity: analysis by sex

|  | **Boys** |  |  |  |  |  | **Girls** |  |  |  |  |  |
| --- | --- | --- | --- | --- | --- | --- | --- | --- | --- | --- | --- | --- |
|  | **Overweight** |  |  | **Obese** |  |  | **Overweight** |  |  | **Obese** |  |  |
|  | OR (95%-CI) | N | n* | OR (95%-CI) | N | n* | OR (95%-CI) | N | n* | OR (95%-CI) | N | n* |
| **Wheeze past year** | |  |  |  |  |  |  |  |  |  |  |  |
| All centres | 1.26 (1.05;1.53) | 4801 | 20 | 1.94 (1.45;2.60) | 3768 | 18 | 0.98 (0.71;1.34) | 4628 | 19 | 1.47 (1.08;2.00) | 3068 | 16 |
| Affluent | 1.44 (1.16;1.79) | 3863 | 14 | 2.09 (1.49;2.93) | 3051 | 13 | 1.07 (0.74;1.55) | 3760 | 14 | 1.63 (1.11;2.40) | 2462 | 12 |
| Nonaffluent | 0.88 (0.62;1.26) | 938 | 6 | 1.44 (0.62;3.34) | 717 | 5 | 0.71 (0.37;1.4) | 868 | 5 | 1.17 (0.67;2.04) | 606 | 4 |
| **Wheeze with exercise** | |  |  |  |  |  |  |  |  |  |  |  |
| All centres | 1.29 (0.98;1.70) | 3519 | 16 | 1.65 (1.04;2.62) | 2956 | 14 | 1.31 (0.95;1.80) | 3542 | 16 | 1.75 (1.14;2.70) | 2044 | 11 |
| Affluent | 1.31 (0.97;1.78) | 2818 | 12 | 1.62 (0.92;2.86) | 2425 | 11 | 1.46 (1.02;2.08) | 2762 | 12 | 1.86 (1.09;3.17) | 1542 | 8 |
| Nonaffluent | 1.19 (0.47;2.99) | 701 | 4 | 1.67 (0.85;3.28) | 531 | 3 | 0.84 (0.35;2.03) | 780 | 4 | 1.67 (0.74;3.78) | 502 | 3 |
| **BHR yes/no** |  |  |  |  |  |  |  |  |  |  |  |  |
| All centres | 0.90 (0.64;1.28) | 2506 | 18 | 1.06 (0.73;1.55) | 2037 | 15 | 1.17 (0.89;1.53) | 2283 | 17 | 1.39 (0.87;2.24) | 1493 | 13 |
| Affluent | 1.08 (0.81;1.43) | 2124 | 14 | 1.07 (0.72;1.58) | 1820 | 13 | 1.21 (0.91;1.61) | 2011 | 14 | 1.43 (0.86;2.38) | 1309 | 11 |
| Nonaffluent | 0.36 (0.12;1.11) | 382 | 4 | 0.98 (0.27;3.63) | 217 | 2 | 0.79 (0.26;2.39) | 272 | 3 | 1.15 (0.31;4.22) | 184 | 2 |
| **Skin prick test** | |  |  |  |  |  |  |  |  |  |  |  |
| All centres | 1.03 (0.86;1.22) | 3930 | 20 | 1.20 (0.86;1.67) | 3168 | 17 | 1.06 (0.88;1.27) | 3831 | 20 | 1.07 (0.76;1.50) | 2961 | 18 |
| Affluent | 1.04 (0.86;1.26) | 3029 | 14 | 1.07 (0.81;1.41) | 2539 | 13 | 1.03 (0.82;1.29) | 2925 | 14 | 1.00 (0.66;1.52) | 2400 | 14 |
| Nonaffluent | 0.94 (0.60;1.47) | 901 | 6 | 2.32 (0.48;11.25) | 629 | 4 | 1.20 (0.77;1.89) | 906 | 6 | 1.23 (0.59;2.55) | 561 | 4 |
| **Total IgE (cutpoint: 4.17kU/l)** | |  |  |  |  |  |  |  |  |  |  |  |
| All centres | 0.88 (0.70;1.11) | 2287 | 15 | 1.03 (0.72;1.47) | 1978 | 15 | 1.05 (0.85;1.31) | 2164 | 15 | 1.15 (0.76;1.73) | 1690 | 13 |
| Affluent | 0.90 (0.71;1.13) | 2147 | 13 | 1.01 (0.70;1.45) | 1908 | 14 | 1.07 (0.86;1.35) | 1997 | 13 | 1.18 (0.78;1.78) | 1622 | 12 |
| Nonaffluent | 0.64 (0.24;1.74) | 140 | 2 | Only one centre left |  |  | 0.83 (0.36;1.93) | 167 | 2 | Only one centre left |  |  |
| **Rhinitis** |  |  |  |  |  |  |  |  |  |  |  |  |
| All centres | 1.13 (0.96;1.34) | 4783 | 21 | 1.16 (0.84;1.59) | 4018 | 19 | 0.89 (0.73;1.08) | 4646 | 20 | 1.20 (0.83;1.74) | 3375 | 17 |
| Affluent | 1.11 (0.92;1.33) | 3808 | 14 | 1.14 (0.79;1.65) | 3308 | 14 | 0.82 (0.63;1.06) | 3698 | 14 | 0.93 (0.65;1.34) | 2764 | 13 |
| Nonaffluent | 1.23 (0.87;1.74) | 975 | 7 | 1.12 (0.43;2.90) | 710 | 5 | 1.10 (0.78;1.55) | 948 | 6 | 2.25 (1.32;3.85) | 611 | 4 |
| **Eczema past year** | |  |  |  |  |  |  |  |  |  |  |  |
| All centres | 1.31 (1.03;1.66) | 4618 | 19 | 1.43 (0.96;2.11) | 3716 | 16 | 1.07 (0.84;1.36) | 4588 | 19 | 1.26 (0.73;2.17) | 3312 | 13 |
| Affluent | 1.48 (1.13;1.94) | 3700 | 13 | 1.62 (1.08;2.43) | 3164 | 12 | 1.07 (0.85;1.35) | 3624 | 13 | 1.40 (0.70;2.80) | 2753 | 9 |
| Nonaffluent | 0.78 (0.41;1.47) | 918 | 6 | 0.99 (0.37;2.62) | 552 | 4 | 0.99 (0.65;1.51) | 964 | 6 | 0.86 (0.41;1.83) | 559 | 4 |
| **Eczema by examination** | |  |  |  |  |  |  |  |  |  |  |  |
| All centres | 2.03 (1.30;3.19) | 2888 | 11 | 1.47 (0.64;3.39) | 1724 | 7 | 1.15 (0.78;1.71) | 3047 | 13 | 1.78 (0.87;3.65) | 1583 | 8 |
| Affluent | 2.07 (1.29;3.33) | 2697 | 9 | 1.50 (0.61;3.65) | 1654 | 6 | 1.16 (0.77;1.76) | 2723 | 10 | 1.87 (0.89;3.94) | 1515 | 7 |
| Nonaffluent | 1.68 (0.38;7.45) | 191 | 2 | Only one centre left |  |  | 1.12 (0.22;5.79) | 324 | 3 | Only one centre left |  |  |
| **FEV1/FVC #** |  |  |  |  |  |  |  |  |  |  |  |  |
| All centres | -0.010 (-0.016;-0.003) | 2736 | 9 | -0.028 (-0.043;-0.012) | 2458 | 9 | -0.007 (-0.015;-0.0002) | 2703 | 9 | -0.018 (-0.036;-0.0007) | 2288 | 9 |
| Affluent | -0.012 (-0.019;-0.004) | 2491 | 7 | -0.022 (-0.036;-0.008) | 2232 | 7 | -0.006 (-0.015;0.003) | 2479 | 7 | -0.012 (-0.022;-0.0008) | 2092 | 7 |
| Nonaffluent | 0.012 (-0.012;0.037) | 245 | 2 | -0.053 (-0.102;-0.005) | 226 | 2 | -0.015 (-0.034;0.003) | 224 | 2 | -0.057 (-0.107;-0.007) | 196 | 2 |

# Adjusted for age and height

* Number of centres

OR: Odds ratios
